# Supplementary material for: Exploiting native forces to capture chromosome conformation in mammalian cell nuclei
Source: Mol Syst Biol. 2016 Dec 9;12(12):891. doi: 10.15252/msb.20167311 (PMC5199122; doi:10.15252/msb.20167311)
Supplement: Supplementary file 1 — Appendix [file MSB-12-891-s001.pdf]

Appendix

Exploiting native forces to capture chromosome conformation in mammalian cell nuclei

Lilija Brant,[1] Theodore Georgomanolis,[1,#] Milos Nikolic,[1,#] Chris A. Brackley,[2] Petros Kolovos,[3] Wilfred van Ijcken,[4] Frank G. Grosveld,[3] Davide Marenduzzo[2] & Argyris Papantonis[1,]*

[1]*Center for Molecular Medicine, University of Cologne, D-50931 Cologne, Germany*
[2]*School of Physics and Astronomy, University of Edinburgh, EH9 3FD Edinburgh, UK*
[3]*Department of Cell Biology, Erasmus Medical Center, 3015 CN Rotterdam, Netherlands*
[4]*Biomics Department, Erasmus Medical Center, 3015 GE Rotterdam, Netherlands*
[#]*These authors contributed equally to this work*

**Corresponding author*: A.P.; argyris.papantonis@uni-koeln.de

This **Appendix** contains:

– Appendix Methods and References (pg. 2-4)

– Figures S1-S24 (pg. 5-37)

– Tables S1-S4 (pg. 38-39)

Appendix Methods and References

Conventional 3C

Conventional 3C/4C experiments were performed following a standard protocol as described previously (Stadhouders *et al*, 2013). In brief, 10^7 cells are fixed in 1% para-formaldehyde (10 min, room temperature) quenched (5 min; RT) and harvested in 0.125 M glycine/PBS. Cell nuclei are isolated in 0.4% NP-40, pelleted and resuspended in the appropriate restriction enzyme buffer supplemented with 0.3% SDS (shaking at 900 rpm for 1h; 37°C). After sequestering SDS with 2% Triton X-100 (shaking at 900 rpm for 1h; 37°C), nuclei are treated overnight with *Apo*I or *Nla*III while shaking (New England Biolabs; 400 U, 37°C). Next, the enzyme is heat inactivated by adding 1.6% SDS (20-25 min, 65°C), nuclei are diluted to 7 ml in ligation buffer supplemented with 1% Triton X-100 to sequester SDS (1h, 37°C), ligated in the presence of 100 units of T4 DNA ligase (Invitrogen; 6-8 h at 16°C), crosslinks are reversed in the presence of proteinase K (65°C, overnight) and then RNase A (30 min, 37°C), and DNA is isolated. This serves as template in 3C-qPCR or 4C-seq performed as described above, using the same primers as for i3C/i4C.

Molecular dynamics simulations

Polymer simulations were performed according to the scheme developed previously (Brackley *et al*, 2016a; 2016b). First, the target region (chr14:53900000-55650000; hg19) is divided into 1-kbp windows, each represented by a polymer "bead". Protein complexes are also represented by simple spheres, which interact attractively with specific beads on the polymer. These are determined using the HUVEC integrated ChromHMM dataset (Hoffman *et al*, 2013) where each 200 bp region of the genome is given a particular chromatin state. We specified three types of bridging proteins, and four types of polymer beads. The first type of protein, denoted “active bridges”, interact strongly with polymer beads representing regions labelled as "active promoters" or "strong enhancers"; these proteins also interact weakly with polymer beads representing HMM regions labelled as "transcriptional transition" or "elongation". The second protein type, “heterochromatin bridges”, interacts weakly with “heterochromatin” regions, and the third type interacts weakly with H3K27me3 “repressed” regions. Since polymer beads cover 1 kbp, it was possible for a bead to cover regions in different states, and so interact with more than one type of protein; beads not overlapping any ChromHMM state do not interact with any bridging proteins. The dynamics of the diffusing polymer and of the protein complexes are simulated using LAMMPS (Plimpton *et al*, 1995) in "Langevin Dynamics" mode; the position of each bead is determined by an equation that describes the potential for interactions between all elements in the system. These potentials include: spring bonds between adjacent beads along the polymer (finite extensible non-linear elastic bonds); angle interactions between triplets of adjacent polymer beads (giving the polymer bending rigidity); steric interactions between all beads to prevent them from overlapping (Weeks-Chandler-Anderson potential); and attractive interactions between protein spheres and the polymer beads (shifted truncated Lennard-Jones potential). We performed 500 independent simulations of this 1750-bead polymer with 40 active, and 80 each of the heterochromatin and H3K27me3-binding complexes. The system extends 150-bead diameters (σ), the persistence length of the polymer is 4σ, and we use

interaction energies of 10 k_BT and 4 k_BT for the strong and weak Lennard-Jones interactions, respectively (with a cut-off of 1.8σ). Each 1-kbp polymer bead has a diameter of 20.8 nm, and each simulation is run for the equivalent of 10.24 sec. We generate 4C-seq profiles or Hi-C maps by averaging over the final conformations of all simulations. Any two polymer beads are said to be in contact if they are separated by less than 2.75σ, and we take the fraction of conformations in which beads are in contact to be the interaction probability. Finally, to compare the simulated profiles with experimental data, we first bin the data and then calculate a Pearson's correlation coefficient for any given pair of profiles.

TALE-iD

We used a plasmid construct encoding a transcription activator–like effector (TALE) DNA-binding domain fused to the LSD1 demethylase, which was shown to bind an enhancer located 50-kbp downstream of the *ZFPM2* TSS that is active in K562 cells (#28 in Mendenhall *et al*, 2013). We replaced the LSD1 domain with the open reading frame of the bacterial DNA adenine methyl-transferase (Dam) gene (and a V5 linker is placed between the TALE and Dam ORFs). This Dam domain has been used to successfully map native binding sites of chromatin-bound factors (DamID; Vogel *et al*, 2006); as a result we named this approach "TALE-iD". We transfect ~3 μg of this construct in $2.5x10^5$ K562 cells using the Nucleofector kit V (Lonza) as per manufacturer's instructions and, in parallel, we also introduce a TALE-DamiD construct with a scrambled DNA-binding domain (Mendenhall *et al*, 2013) or with one where the Dam ORF is disrupted to inactivate the methylase. 48 h post-transfection, cells are harvested and total genomic DNA is isolated, digested with 1 unit/μg *Dpn*I for ~2 h, and purified using a DNA purification kit (Zymo Research). Finally, qPCR is applied using primers that encompass *Dpn*I sites in the *ZFPM2* locus (sequences available upon request). Lower amplification levels (i.e., higher Ct values) signify over-background methylation at the respective site, and enrichments (1/ΔΔCt) are calculated after normalizing for the amplification efficiency of the primer pair (on K562 genomic DNA), as well as for "basal" *Dpn*I digestion of DNA in untransfected cells.

T2C/Hi-C and data analysis

T2C/iT2C was performed in two independent replicates as described (Kolovos *et al*, 2014). In brief, ~10 million HUVEC nuclei were crosslinked in 1% paraformaldehyde (for conventional T2C) or processed as for i3C (for iT2C). Chromatin was digested using *Apo*I (New England Biolabs), and ligated under conditions that allowed nuclei to remain intact. Then, ligated DNA was reduced in size by a combination of *Dpn*II digestion and sonication, and sequenced to >50 million reads per sample on a HiSeq2000 platform (Illumina). Raw data were mapped to the reference genome (hg19) and analyzed via custom R scripts (see below). Finally, data binned to achieve 10-, 5-, and 1.5-kbp resolution were visualized in 2D interaction plots. PE-SCAN (de Wit *et al*, 2013) was used to generate interaction enrichment plots. TAD/subTAD boundaries were called via the "directionality index" using a Hidden Markov Model (as described in Dixon *et al*, 2012). Note that IMR90 iT2C was performed in two technical replicates that were merged.

iHi-C was performed using 25 million HUVECs simply by incorporating labelling of DNA ends by biotin-dATP after *Dpn*II chromatin digestion in the flow of the i3C protocol. Then, DNA ends

(now blunt) are ligated in intact nuclei, and DNA isolated and sonicated to ~800 bp. Finally, biotinylated 3C junction are captured on streptavidin beads, washed to remove non-captured DNA, amplified for 6 PCR cycles to add sequencing linkers, and sequenced on a HiSeq4000 platform (Illumina) to 300 million read pairs (75 bp-long). The resulting reads were then mapped to the reference genome (hg19) iteratively (to ensure maximum recovery of uniquely mapped pairs) using BWA (Li & Durbin, 2010), duplicates were removed (http://picard.sourceforge.net/), and the output converted into BEDPE format (Quinlan *et al*, 2010). Then, custom R scripts were used to bin the genome into non-ovelapping bins, to assign reads to bins, and to normalize read counts to library size. Then, the HiTC package (Servant *et al*, 2012) was used to correct matrices for biases in genomic features (Yaffe & Tanay, 2011) and to visualize 2D heat maps.

Brackley CA, Johnson J, Kelly S, Cook PR, Marenduzzo D (2016b) Binding of bivalent transcription factors to active and inactive regions folds human chromosomes into loops, rosettes and domains. *Nucleic Acids Res* 44: 3503-3512

Caudron-Herger M, Cook PR, Rippe K, Papantonis A (2015) Dissecting the nascent human transcriptome by analysing the RNA content of transcription factories. *Nucleic Acids Res* 43: e95

Guelen L, Pagie L, Brasset E, Meuleman W, Faza MB, Talhout W, Eussen BH, de Klein A, Wessels L, de Laat W, van Steensel B (2008) Domain organization of human chromosomes revealed by mapping of nuclear lamina interactions. *Nature* 453: 948-51

Hoffman MM, Ernst J, Wilder SP, Kundaje A, Harris RS, Libbrecht M, Giardine B, Ellenbogen PM, Bilmes JA, Birney E, Hardison RC, Dunham I, Kellis M, Noble WS (2013) Integrative annotation of chromatin elements from ENCODE data. *Nucleic Acids Res* 41: 827-841

Németh A, Conesa A, Santoyo-Lopez J, Medina I, Montaner D, Péterfia B, Solovei I, Cremer T, Dopazo J, Längst G (2010) Initial genomics of the human nucleolus. *PLoS Genet* 6: e1000889

Plimpton S (1995) Fast parallel algorithms for short-range molecular dynamics. *J Comput Phys* 117: 1-19

Quinlan AR, Hall IM (2010) BEDTools: a flexible suite of utilities for comparing genomic features. *Bioinformatics* 26: 841-842

Sato T, SuyamaM (2015) ChromContact: A web tool for analyzing spatial contact of chromosomes from Hi-C data. *BMC Genomics* 16: 1060

Servant N, Lajoie BR, Nora EP, Giorgetti L, Chen CJ, Heard E, Dekker J, Barillot E (2012) HiTC: exploration of high-throughput 'C' experiments. *Bioinformatics* 28: 2843-2844

The ENCODE Project Consortium (2012) An integrated encyclopedia of DNA elements in the human genome. *Nature* 489: 57-74

Yaffe E, Tanay A (2011) Probabilistic modeling of Hi-C contact maps eliminates systematic biases to characterize global chromosomal architecture. *Nat Genet* 43: 1059-1065

Appendix Figures

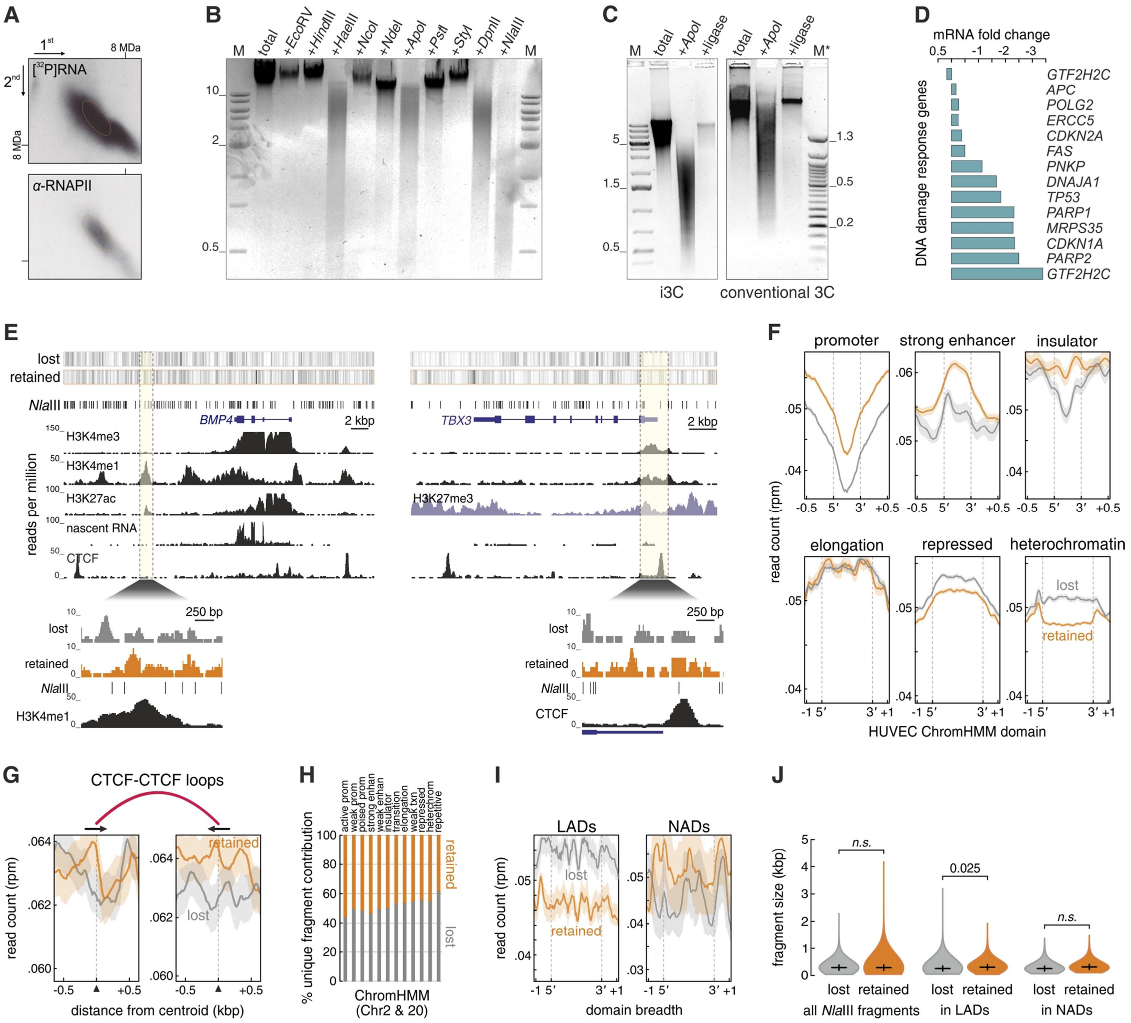

Figure S1. Features of the i3C approach.

(**A**) Quantitative retention of HUVEC transcriptional activity in PB (adapted from Caudron-Herger *et al*, 2015). HUVECs harvested in PB and permeabilized with saponin were used in a nuclear run-on in the presence of [^{32}P]UTP (*top*), and for immunoblotting the largest subunit, RPB1, of RNA polymerase II (*bottom*) in 2D native gel electrophoresis. Dotted ovals denote gel regions rich in nascent transcripts and RPB1; the migration of an 8-MDa marker is also indicated.

(**B**) Digestion efficiency in PB. A range of commercially-available restriction endonucleases were tested for cutting chromatin in HUVEC nuclei in PB+0.4% NP-40. Size markers (M) are shown.

(**C**) Chromatin digestion and re-ligation efficiency. i3C and conventional 3C were performed in HUVECs in parallel. Electrophoretic profiles of chromatin cut (“+*Apo*I”) and re-ligated (“+ligase”) in each procedure is compared to that of total cell DNA. Size markers (M and M*) are shown.

(**D**) Bar plots show fold changes in mRNA levels of DNA damage response genes comparing whole-cell RNA-seq data to that from nuclei digested with DNase I (Caudron-Herger *et al*, 2015).

(**E**) Browser views of genomic coverage for different i3C chromatin fractions. HUVEC DNA isolated from the “lost” (step 3 in Fig. 1A) and “retained” chromatin fractions (step 4 in Fig. 1A) of the i3C approach was sequenced to ~40 million reads and mapped to the genome (hg19). Typical loci are shown, where heatmaps (*top*) illustrate read coverage in each fraction aligned to ENCODE ChIP-seq data for an active (*BMP4*) and an inactive gene locus (*TBX3*). *Magnifications*: detailed coverage views in the highlighted regions.
(**F**) Line plots show raw read coverage (in reads per million) of the “retained” (*orange line*) or the “lost” fraction (*grey line*) along regions variably annotated in HUVEC ChromHMM segmentation.
(**G**) Line plots show raw read coverage (in rpm) of the “retained” (*orange line*) or the “lost” fraction (*grey line*) around CTCF sites (“centroid”) that contribute to loop formation in HUVECs (positions obtained from Rao *et al*, 2014).
(**H**) Bar plot showing the per cent contribution of different HUVEC chromHMM features in reads unique to the “lost” or the “retained” fraction (data from exemplary chromosomes 2 and 20).
(**I**) Line plots show raw read coverage (in rpm) of the “retained” (*orange line*) or the “lost” fraction (*grey line*) along lamin- (LADs) and nucleolus-associated domains (NADs) mapped in fibroblast cells (regions taken from Guelen *et al*, 2008 and Nemeth *et al*, 2010, respectively).
(**J**) Bean plots show the size distribution of *Nla*III fragments in the “retained” (*orange*) or the “lost” fraction (*grey*) for all data, LADs, and NADs. *P*-values were calculated using a two-tailed unpaired Student’s t-test.

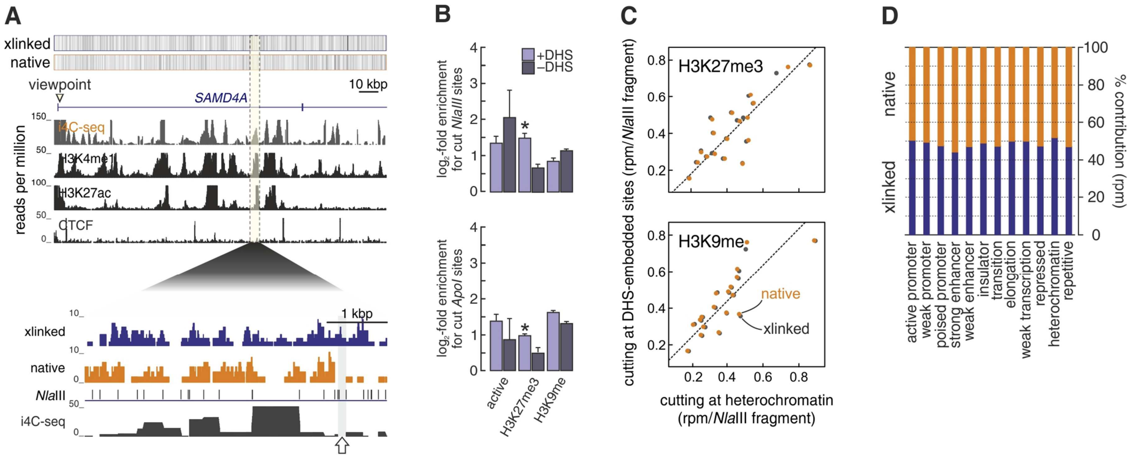

Figure S2. Comparison of i3C and conventional 3C DNA templates.

(**A**) Coverage of *Nla*III fragments by chromatin digested under crosslinked ("xlinked") or native conditions. HUVEC DNA isolated from step 2 of the i3C/3C procedure (see Fig. 1A) was sequenced to ~40 million reads and mapped to the genome (hg19). A typical locus is shown, where heatmaps (*top*) illustrate read coverage in each fraction aligned to i4C-seq and ChIP-seq data for the *SAMD4A* locus. *Magnification*: detailed coverage view in the highlighted region. A fragment missing from the i3C template that also lacks i4C-seq signal is indicated (*open arrow*).

(**B**) Log_2-enrichment for cut *Apo*I (*left*) or *Nla*III sites (*right*) embedded (*purple*) or not (*grey*) in DNase hypersensitive (DHS) regions. Data (±SEM) from two replicates of two sites in active, Polycomb-marked (H3K27me3) or heterochromatic (H3K9me1) regions are shown. *P<0.05; unpaired two-tailed Student's t-test (n=4).

(**C**) Scatter plots compare the number of reads contained in DHS versus heterochromatic regions per chromosome (H3K9me1; *top* – H3K27me3; *bottom*) after cutting with *Nla*III in the presence (*grey*) or absence of crosslinking (*orange*). Essentially no differences are seen.

(**D**) Relative representation of chromatin features in HUVEC chromatin digested with *Nla*III under crosslinked ("xlinked") or native conditions. The composite bar plot shows per cent contribution of different chromatin features (using HUVEC ENCODE ChromHMM data; Hoffman *et al*, 2013) in the two samples.

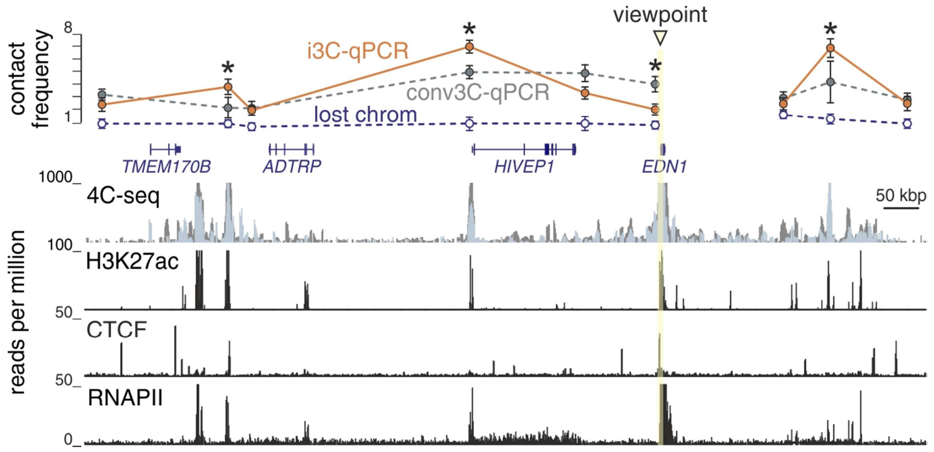

Figure S3. i3C-qPCR implemented in the extended *EDN1* locus on chromosome 6.

The contact frequency (±SD; n=2) between the *EDN1* TSS (“viewpoint”; *triangle*) and 9 segments was assessed using i3C (*orange*), conventional 3C (*grey*) coupled to qPCR, or i3C performed on the “lost” chromatin fraction (*blue*; see Fig. 1A). Interacting and non-interacting segments were selected based on two replicates (*dark/light grey*) of conventional 4C-seq data from the same viewpoint. ENCODE ChIP-seq data (ENCODE, 2012) from HUVECs are also shown. *P<0.05; two-tailed unpaired Student’s t-test (n=2).

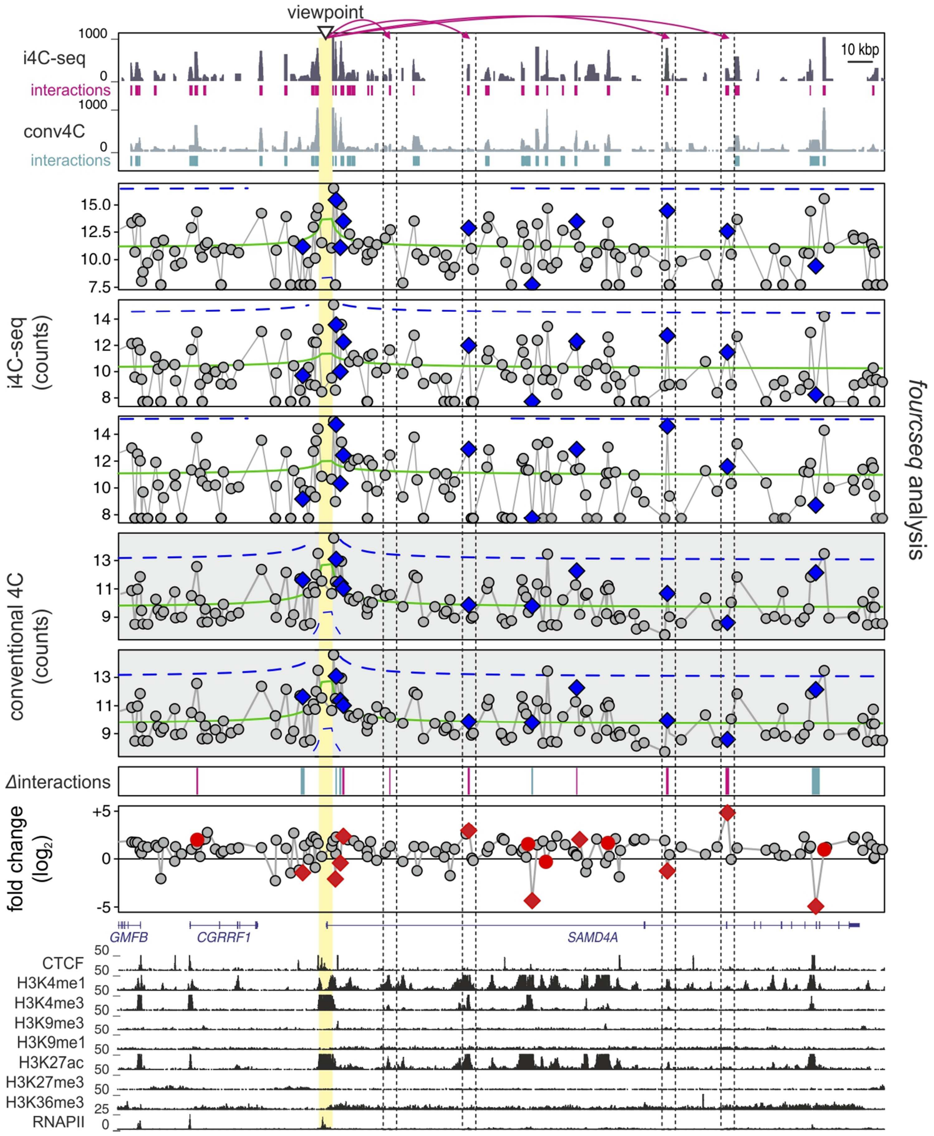

Figure S4. Differential analysis of i4C and conventional 4C data.

FourCSeq (Klein *et al*, 2015) was used to perform differential analysis of contacts between i4C-seq (*white boxes*) and conventional 4C-seq replicates (*grey boxes*) produced in HUVECs using *Apo*I and the *SAMD4A* TSS as viewpoint (*triangle*). Some differences (“*Δ*interactions”) specific to either i4C (*magenta*) or conventional 4C (*green*) are detected, and three specific to i4C are highlighted (*dotted rectangles*). HUVEC ENCODE ChIP-seq data are shown aligned below.

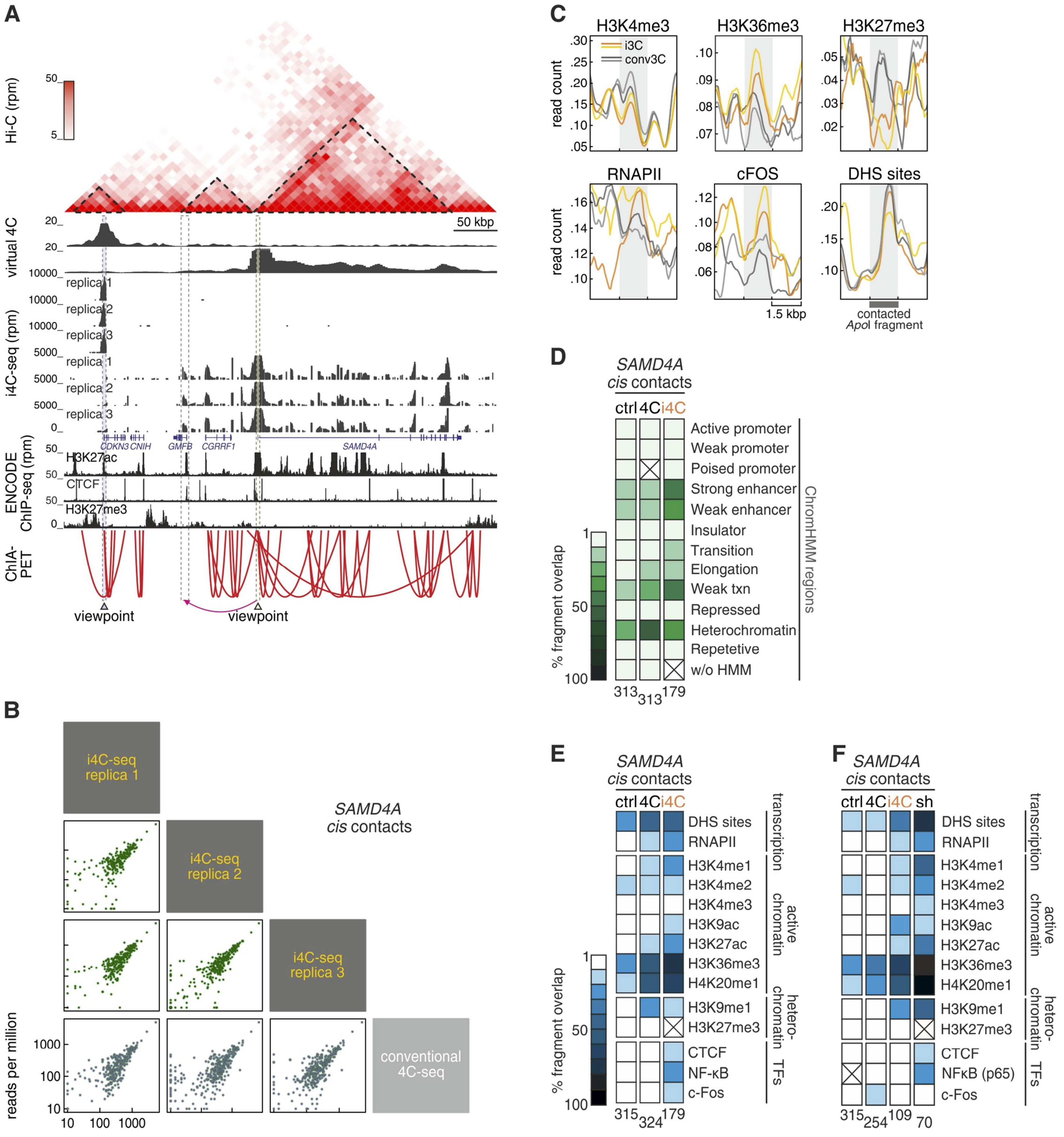

Figure S5. i4C-seq implemented in the gene-rich *SAMD4A* locus on chromosome 14.
(**A**) Three independent i4C-seq replicates were generated in HUVECs using *Apo*I and the *CDKN3* or *SAMD4A* TSSs as viewpoints (*triangles*). i4C interactions in the 0.5 Mbp around the viewpoints are shown aligned to RefSeq gene models and ENCODE ChIP-seq data. Hi-C data from HUVECs (10-kbp resolution; from Rao *et al*, 2014) is used to outline *CDKN3* and *SAMD4A* TADs (*dotted triangles*; from Dixon *et al*, 2012), and to generate "virtual 4C" profiles for each viewpoint (using ChromContact; Sato *et al*, 2015). RNA polymerase II-driven ChIA-PET interactions (Papantonis *et al*, 2012) are also shown below. The arrow at the bottom (*magenta*) indicates a contact not seen by virtual 4C or ChIA-PET.

(**B**) Pairwise correlation plots for the *cis*-interactions of three i4C versus one conventional 4C replicate. All Spearman's correlation coefficients calculated were >0.75.
(**C**) Line plots show raw read coverage of ENCODE ChIP-/DHS-seq data along all *SAMD4A cis*-contacts (±1.5 kbp) from two i4C- (*orange/yellow*) and 4C-seq replicates (*dark/light gray*).
(**D**) The heat map shows the fraction of i4C-/4C-seq *SAMD4A cis*-contacts (*Apo*I fragments with >100 rpm) that overlap the different chromatin elements in HUVECs. The overlap of randomly-shuffled ("ctrl") fragments serves as a control. The numbers below each heat map denote the number of *Apo*I fragments analyzed per dataset.
(**E**) As in panel D, but showing the fraction of i4C-/4C-seq *SAMD4A cis*-contacts (*Apo*I fragments with >100 rpm) overlapping DHS-/ChIP-seq peaks.
(**F**) As in panel E, but showing the fraction of unique or shared ("sh") i4C-/4C-seq *SAMD4A cis*-contacts (*Apo*I fragments with >100 rpm) overlapping DHS-/ChIP-seq peaks.

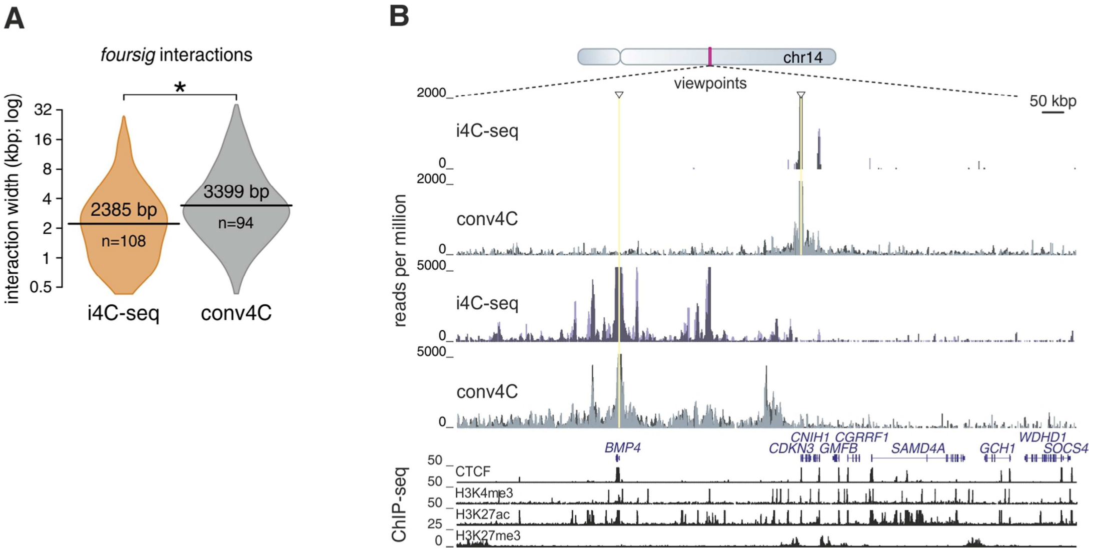

Figure S6. i4C-seq and conventional 4C implemented in loci on chromosome 14..

(**A**) Violin plots show the distribution of *cis*-contact width (*Apo*I fragments) in i4C-seq (*orange*; 3 replicates) and conventional 4C experiments (*right*; 2 replicates). Contacts were identified using *foursig* and the TSS of *SAMD4A* as a viewpoint. Their number (n) and mean width (in bp) are also indicated in each plot.

(**B**) i4C-seq (*purple shades*) and conventional 4C (*grey shades*) were performed side-by-side in HUVECs, using *Apo*I and the *CDKN3/BMP4* TSSs as viewpoints (*triangles*); profiles from two replicates are shown overlaid. The browser view shows interactions in a >1-Mbp region aligned to RefSeq gene models and HUVEC ENCODE ChIP-seq data.

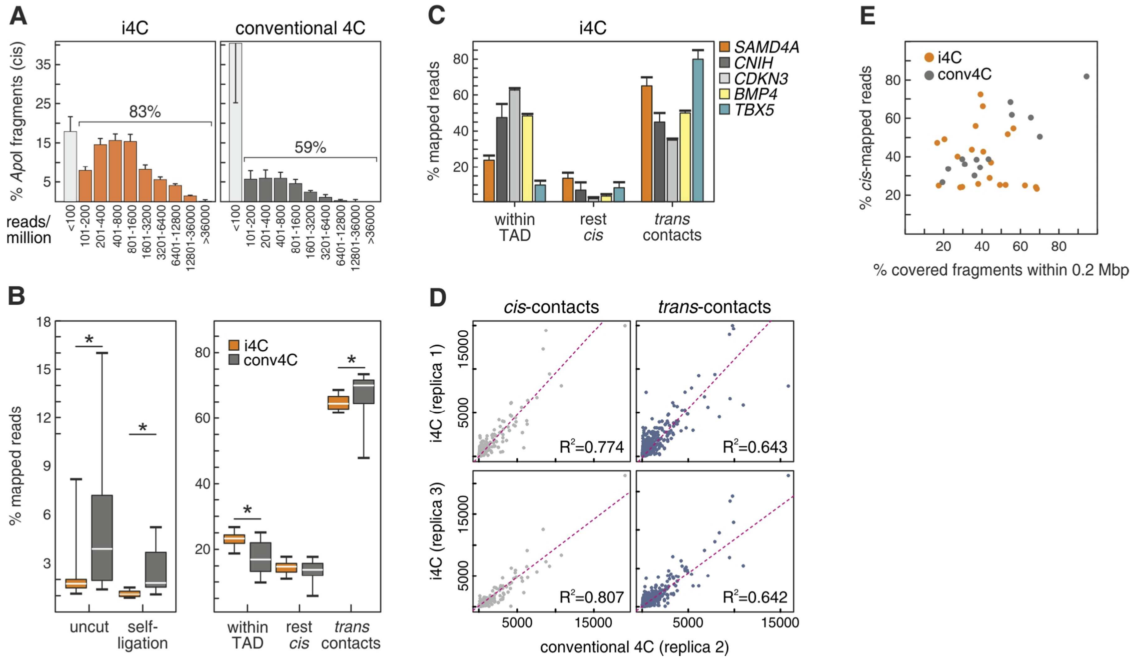

Figure S7. Comparison of attributes in i4C and conventional 4C.

(**A**) Bar plots showing the per cent of all *cis*-contacts (*Apo*I fragments; ±SD) from i4C-seq (*left*; 3 replicates) and 4C-seq experiments (*right*; 2 replicates) binned according to read coverage.

(**B**) Box plots showing the per cent of mapped reads that correspond to uncut and self-ligated fragments (*left*) or to fragments mapping within, outside or in *trans* to the *SAMD4A* TAD (*right*). *: significantly different mean; *P*<0.05, two-tailed unpaired Student's *t*-test.

(**C**) Bar plots showing the per cent of mapped reads that fall within, outside or in *trans* to the TAD of each of the *SAMD4A*, *CNIH*, *CDKN3*, *BMP4*, and *TBX5* viewpoints in HUVECs.

(**D**) Scatter plots correlate that read distribution of two i4C to one conventional 4C replica for both *cis*- and *trans*-contacts. Spearman's correlation coefficients (R^2) are shown for each plot.

(**E**) Scatter plot showing the per cent of total mapped reads in *cis* versus local fragment coverage in the 0.2 Mbp around each viewpoint for i4C/4C experiments performed with *Apo*I or *Nla*III. Reads from self-ligation and uncut products were not included in this analysis (as advised in van de Werken *et al*, 2012), and fragment coverage in i4C data was corrected for the actual number of fragments retained (~55% of total fragments).

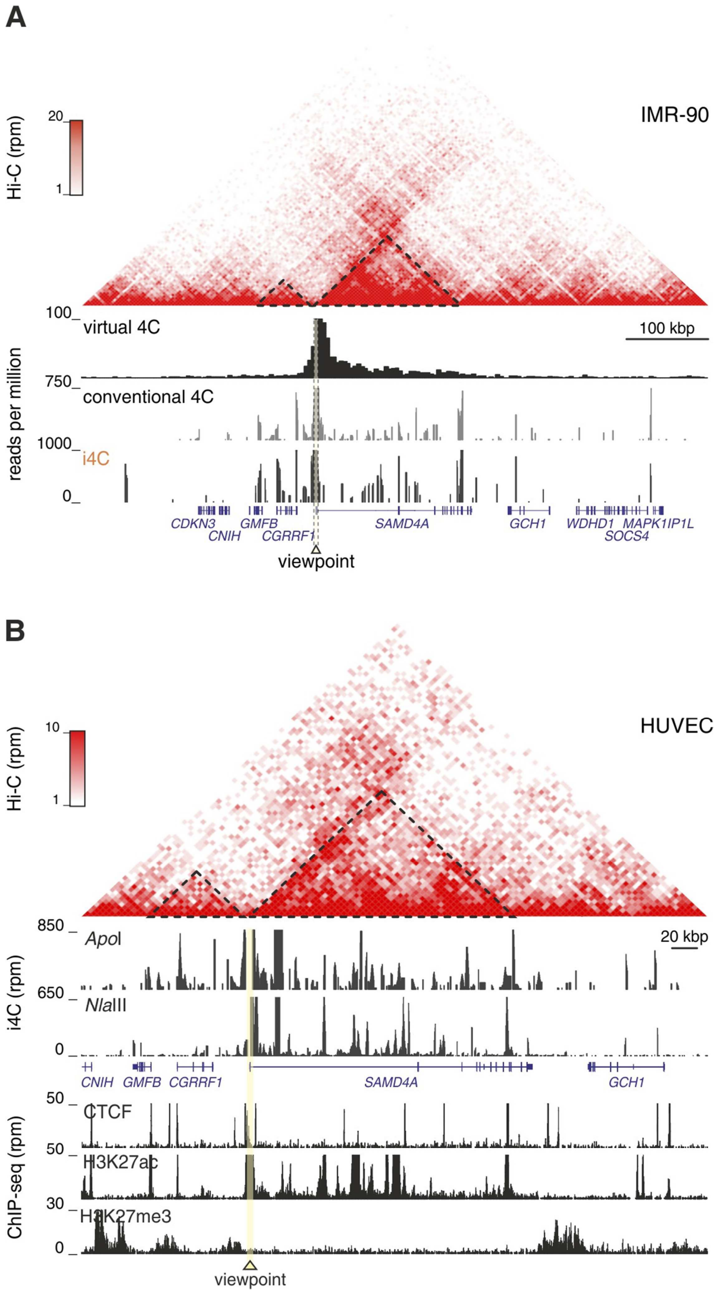

Figure S8. i4C-seq in the *SAMD4A* locus using different cell types and enzymes.
(**A**) i4C-seq was performed in IMR90s using the *SAMD4A* TSS as a viewpoint (*triangle*), and *Apo*I. A genome browser view of ~0.8-Mbp region on chr14 is shown aligned to RefSeq gene models and conventional 4C-seq data. Hi-C data from IMR-90 cells (5-kbp resolution; Rao *et al*, 2014) is used to outline TADs (*dotted triangles*), and to generate a virtual 4C profile for *SAMD4A*.
(**B**) As in panel A, but performed in HUVECs cells using either *Apo*I or *Nla*III. The genome browser view focuses on a 0.5-Mbp region of chromosome 14.

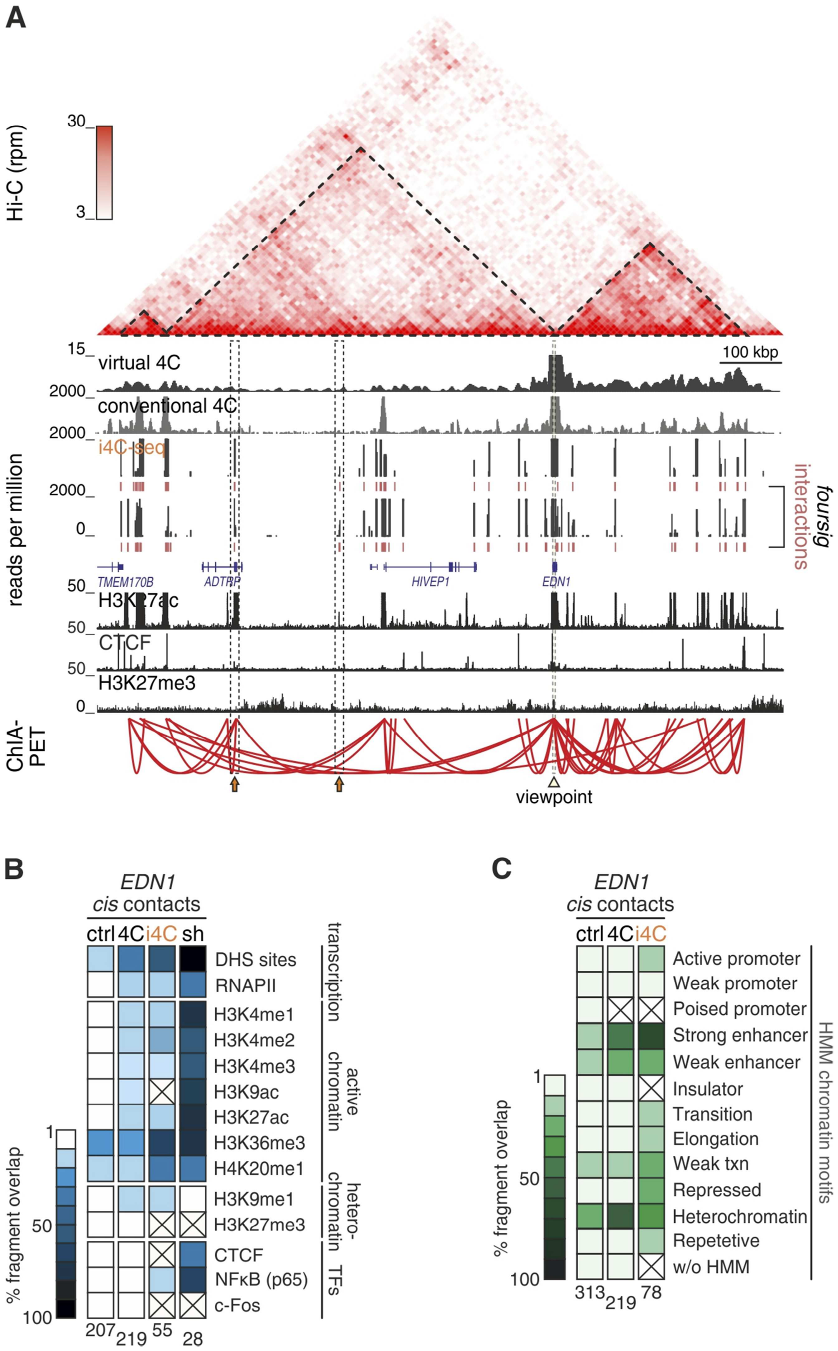

Figure S9. i4C-seq implemented in the gene-poor human *EDN1* locus.
(**A**) i4C/conventional 4C was performed in HUVECs using the *EDN1* TSS as a viewpoint (*triangle*) and *Apo*I. A genome browser view of ~1.5-Mbp on chromosome 6 is shown aligned to RefSeq gene models, HUVEC ENCODE ChIP-, and 4C/i4C-seq data. Hi-C (Rao *et al*, 2014) was used to outline TADs (*dotted triangles*) and to generate virtual 4C for *EDN1*, and ChIA-PET interactions in

the locus (Papantonis *et al*, 2012) are also shown (*bottom*). Two exemplary *EDN1* contacts to H3K27ac-marked regions that are only seen using i4C are indicated by arrows (*orange*).
(**B**) Heat map shows the fraction of unique or shared ("sh") i4C/4C *cis*-contacts (*Apo*I fragments with >100 rpm) overlapping DHS-/ChIP-seq peaks. Randomly-shuffled ("ctrl") fragments serve as a control. The numbers below each heat map denote the number of *Apo*I fragments analyzed per dataset.
(**C**) As in panel B, but for the fraction of *cis*-contacts overlapping HUVEC ChromHMM segments.

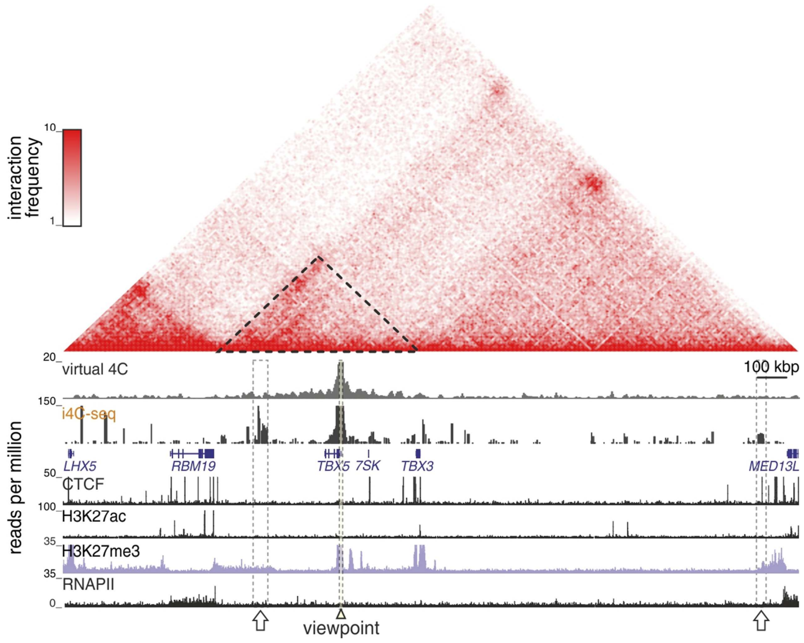

Figure S10. i4C-seq implemented in the heterochromatinized *TBX5* locus.

i4C-seq was performed in HUVECs using the *TBX5* TSS as a viewpoint (*triangle*) and *Nla*III. A genome browser view of a >1.5-Mbp region on chromosome 12 is shown aligned to RefSeq gene models and ENCODE ChIP-seq data. Hi-C data from HUVECs (5-kbp resolution; Rao *et al*, 2014) is used to outline TADs (*dotted triangles*) and generate a virtual 4C profile for *TBX5*. Two strong interactions between H3K27me3-marked regions that are not seen using conventional data are denoted (*arrows*).

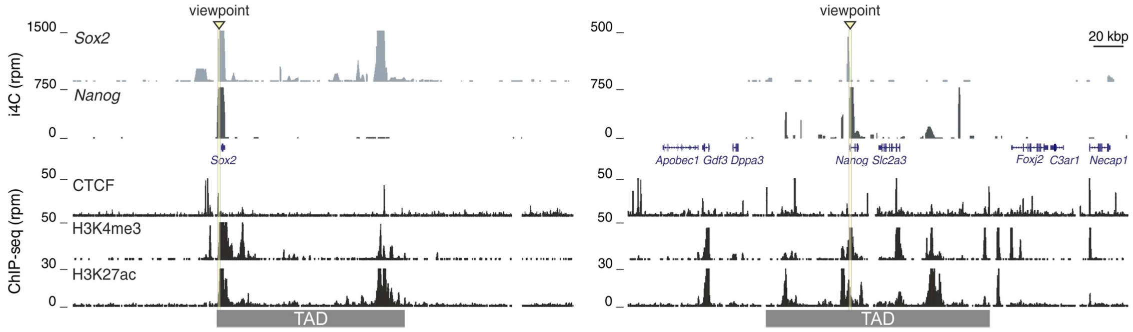

Figure S11. i4C-seq in the *Sox2* and *Nanog* loci in mouse embryonic stem cells.
The CTCF peak proximal to the *Sox2* promoter (on chr 3; *left*) and the *Nanog* TSS (on chr 6; *right*) were used as viewpoints (*triangles*) in i4C performed in mESCs using *Apo*I. Their interactions profiles, aligned to publicly-available ChIP-seq data, confirm previously-recorded contacts within their respective TADs, as well as strong reciprocal interactions (see de Wit *et al*, 2013).

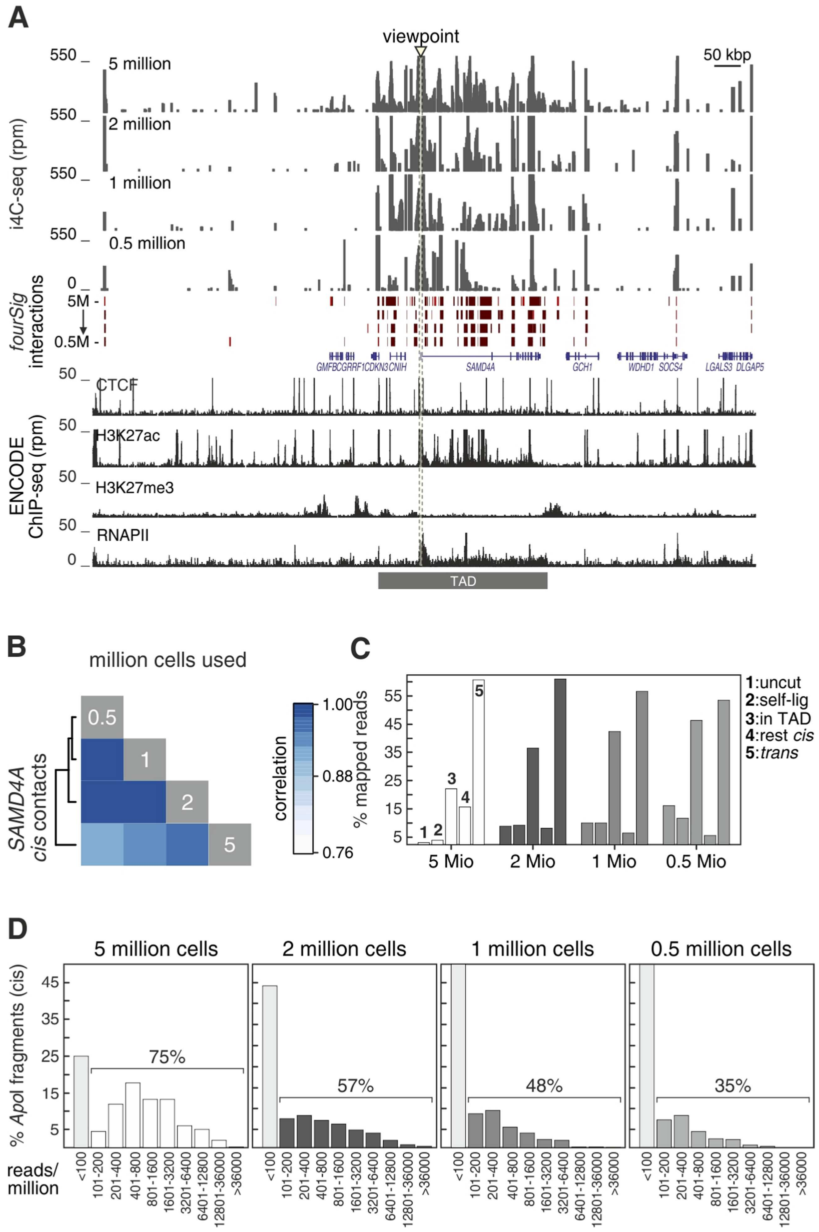

Figure S12. i4C-seq implemented using low counts of isolated nuclei.

(**A**) i4C-seq is performed in HUVECs using the *SAMD4A* TSS as a viewpoint (*triangle*), *Apo*I, and 5-0.5 million nuclei as input. Browser views of i4C data in a 1-Mbp region on chromosome 14 is shown aligned to Refseq gene models and ENCODE ChIP-seq data and the *SAMD4A* TAD (*grey rectangle*). FourSig was used to call significant interactions per profile (*brown*/*orange boxes*).

(**B**) The heat map shows the similarity of all *SAMD4A cis*-contacts between i4C-seq experiments from panel A; Spearman's correlation coefficients were calculated and are shown.

(**C**) Bar plots showing per cent of mapped reads in uncut (*1*), self-ligated (*2*), TAD-contained (*3*), or not (*4*), and *trans* fragments (*5*). Low cell counts show increased uncut/self-ligated fragments.

(**D**) Bar plots show the per cent of *cis*-contacts (*Apo*I fragments) from the i4C experiments in panel A binned according to read coverage. Decreasing the starting material results in increased numbers of fragments with low coverage.

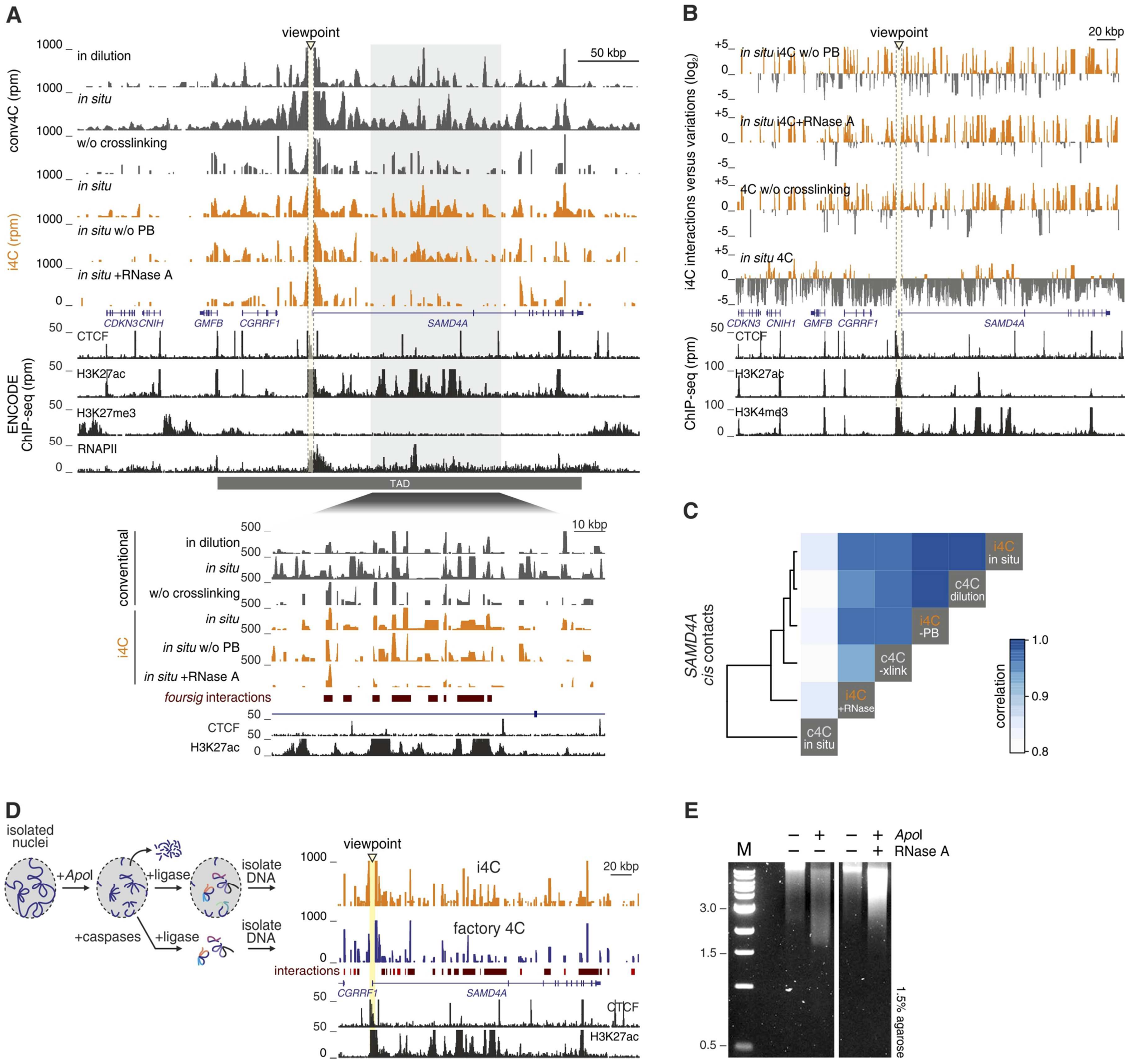

Figure S13. i4C-seq implemented with and compared to different conditions and treatments.

(**A**) i4C-seq (*orange tracks*) was performed side-by-side with conventional 4C (*dark grey tracks*) in HUVECs using the *SAMD4A* TSS as a viewpoint (*triangle*), *Apo*I, and variable conditions and treatments. *From top to bottom*: typical 4C-seq (Stadhouders *et al*, 2013), 4C-seq following the *in situ* Hi-C protocol (Rao *et al*, 2014) with or without crosslinking, the i4C-seq protocol described here, and i4C where nuclei were pretreated with 30 µg RNase A. A genome browser view of a 1-Mbp region on chromosome 14 and all i4C/4C variations is shown aligned to RefSeq gene models, ENCODE ChIP-seq, and the *SAMD4A* TAD (*grey rectangle*). *Magnification*: contacts at the intronic *SAMD4A* enhancer cluster.

(**B**) Log_2 fold change in interaction signal between i4C applied to the SAMD4A TSS (as in panel A) and (*from top to bottom*) i4C without PB, i4C pretreated with RNase A, conventional 4C without crosslinking, and conventional 4C performed using the *in situ* protocol. HUVEC ENCODE ChIP-seq data are also shown aligned to the locus (*below*).

(**C**) The heat map illustrates the similarity of *SAMD4A* i4C *cis*-contacts from the different experimental variations shown in panel A based on Spearman's correlation coefficients.
(**D**) *Left*: an overview of the "factory 4C" procedure (based on "transcription factory" isolation using group-III caspases; see Melnik *et al*, 2011) compared to the i4C procedure. *Right*: i4C (*orange*) and factory 4C (*blue*) contact profiles generated using *Apo*I and the *SAMD4A* TSS as a viewpoint (*triangle*) are aligned to ENCODE ChIP-seq data, and to interactions called by fourSig.
(**E**) Agarose gel electrophoresis profiles of uncut and the *Apo*I-digested "lost" chromatin fraction from HUVECs that were (right) or were not (left) treated with RNase A. Treatment results in ~1.5-fold more chromatin released from the nuclei (quantified using a Qubit device). The sizes of three bands (in kbp) of the molecular marker (M) are indicated.

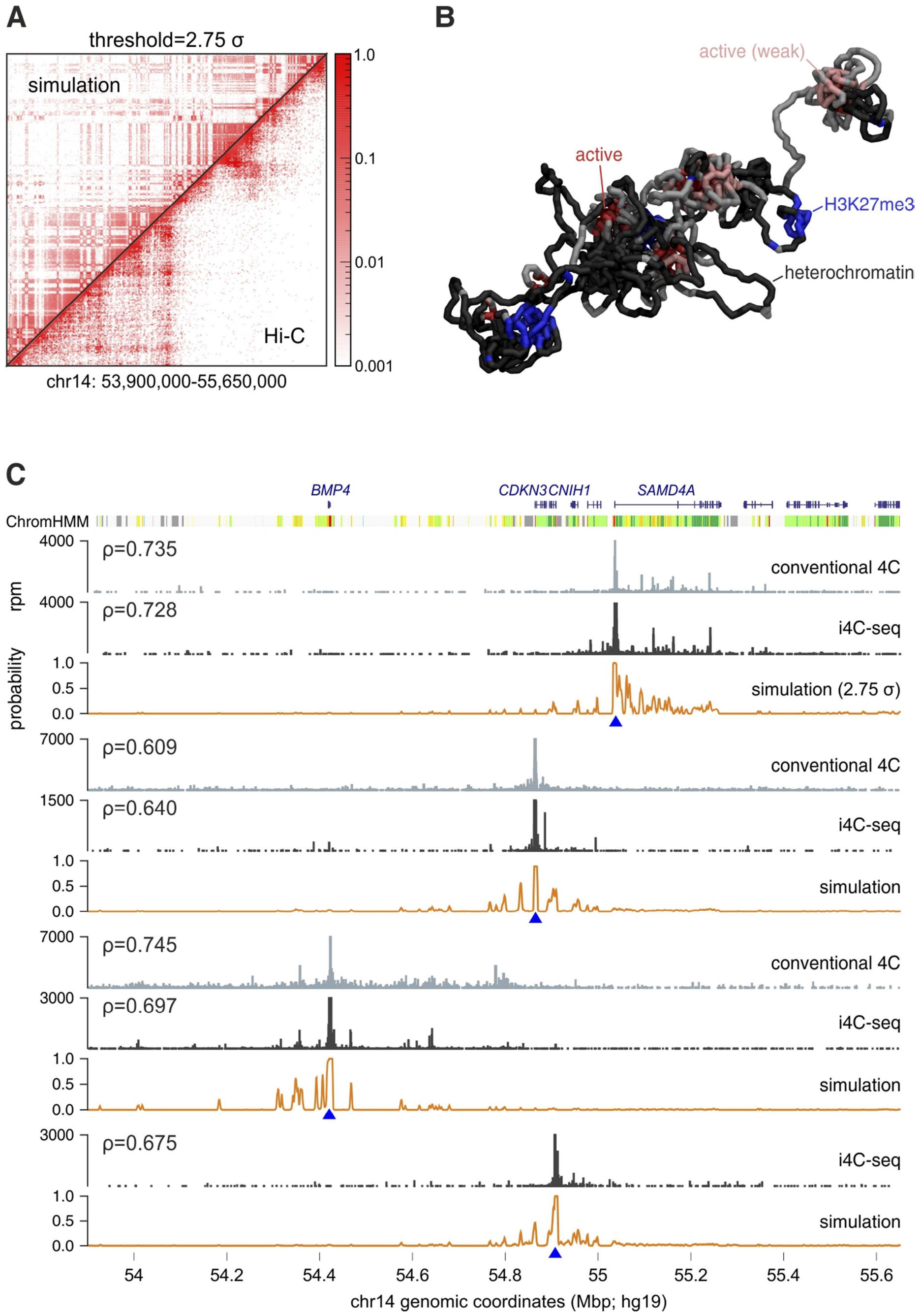

Figure S14. Simulations of spatial contacts in a 2.8-Mbp locus on chromosome 14.
(**A**) Heat map comparing interactions predicted *in silico* (using a 2.75 σ interaction cutoff; *left*) to those seen by Hi-C (at 5-kbp resolution, Rao *et al*, 2014; *right*). The color scale indicates contact probability in the simulated data.

(**B**) 3D visualization of the simulated folding of this 2.8-Mbp locus (one of 500 independently-generated structures). Stretches denoted as transcriptionally-active (*red*), weakly-active (*pink*), Polycomb-bound (*blue*), or heterochromatic (*black*) in ChromHMM data are indicated.
(**C**) Interactions by the *SAMD4A, CKDN3, CNIH,* and *BMP4* TSSs detected using conventional 4C-seq (*grey*), i4C-seq data (*black*), or predicted by simulations (*orange*) are shown aligned to gene models (*blue*), and the ChromHMM partitioning. Pearson's correlation coefficients (ρ) are also shown to facilitate comparison.

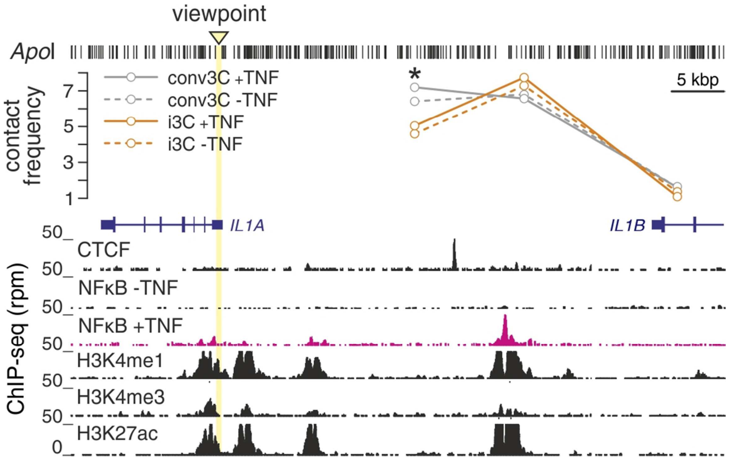

Figure S15. i3C-qPCR implemented in a TNF-responsive locus in HUVECs.

TSS prelooping (±SD; n=2) of the TNF-responsive *IL1A* gene (“viewpoint”; *triangle*) to distal enhancer elements (Jin *et al*, 2013) was assessed using i3C (*orange*) or conventional 3C (*grey*) coupled to qPCR. Data are presented aligned to ENCODE and NF-κB (*magenta*; Papantonis *et al*, 2012) ChIP-seq data in the locus. *$P<0.05$; two-tailed unpaired Student’s t-test (n=2).

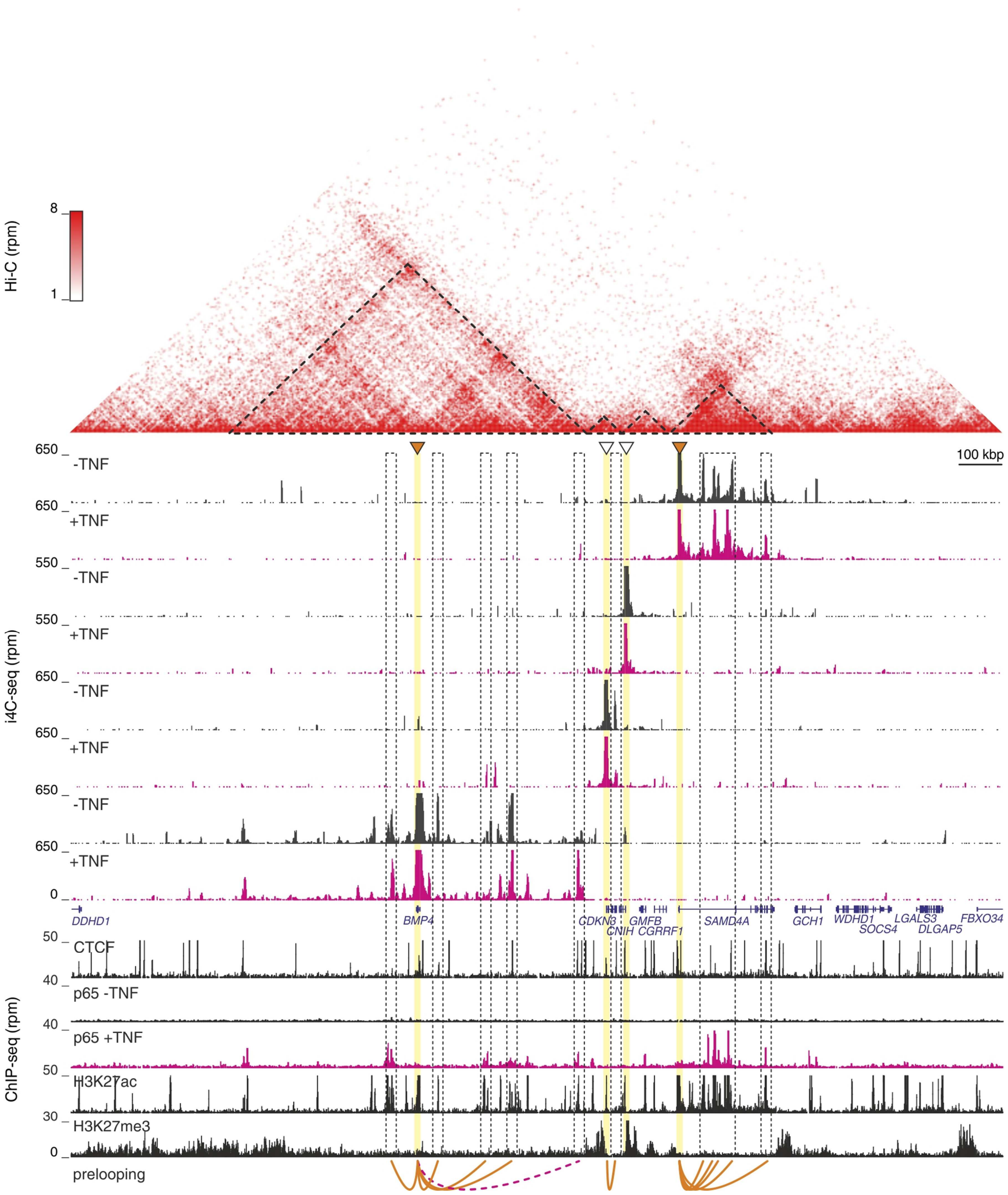

Figure S16. i4C interactions are confined within TADs and describe prelooping.
i4C-seq was performed in HUVECs ±TNF using *Nla*III and the TSSs of *BMP4*, *CDKN3*, *CNIH*, and *SAMD4A* as viewpoints (*triangles*). Interaction profiles are shown aligned to Hi-C (*top*; Rao *et al*, 2014) and HUVEC ENCODE ChIP-seq data (*below*). Examples of prelooped and changing i4C interactions by the *BMP4*, *CDKN3*, and *SAMD4A* TSSs to enhancers are indicated (*rectangles* and *solid/dotted lines*).

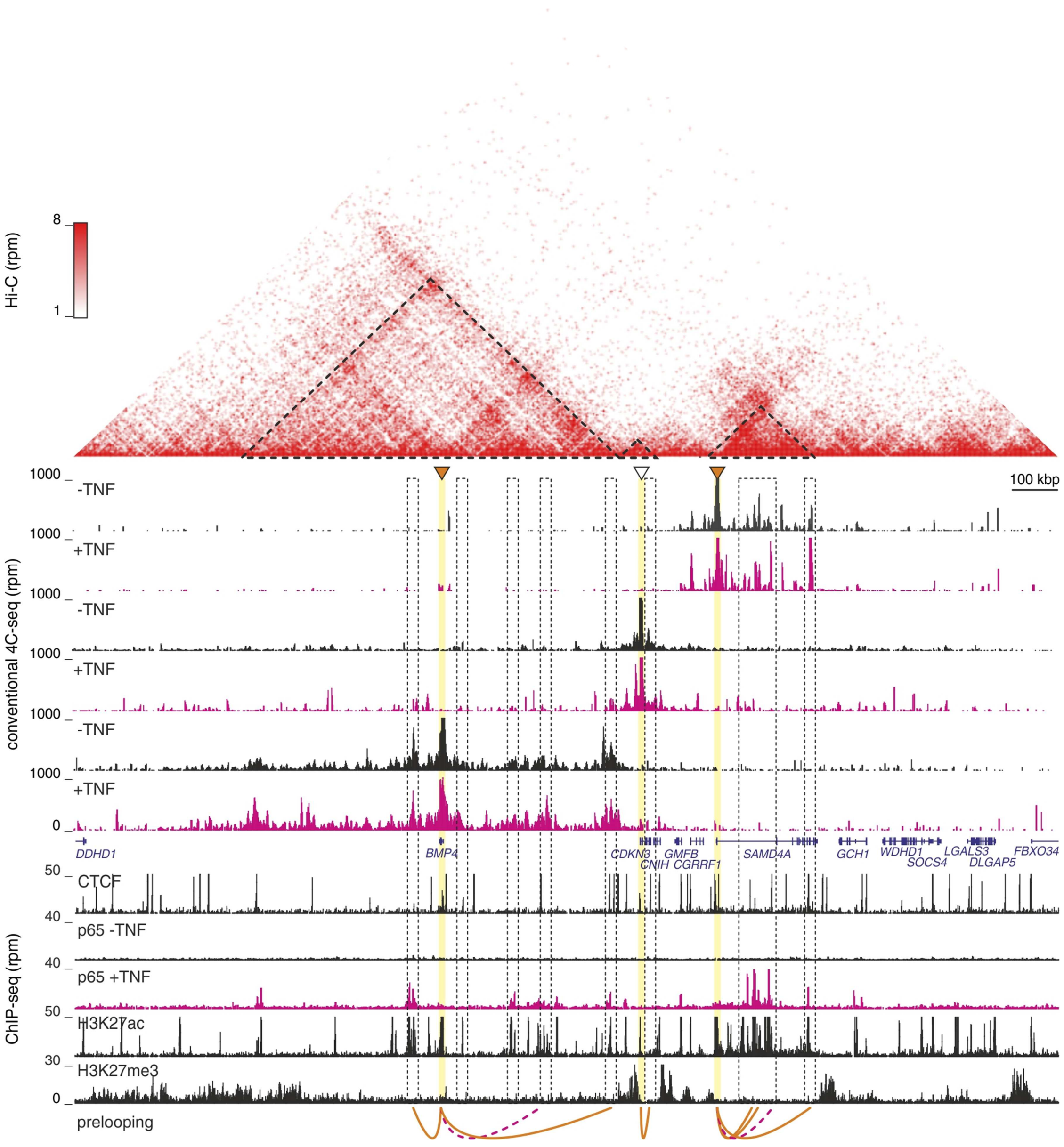

Figure S17. Conventional 4C interactions upon TNF stimulation on chromosome 14.
i4C-seq was performed in HUVECs ±TNF using *Nla*III and the TSSs of *BMP4*, *CDKN3*, and *SAMD4A* as viewpoints (*triangles*). Interaction profiles are shown aligned to Hi-C (*top*; Rao *et al*, 2014) and HUVEC ENCODE ChIP-seq data (*below*). Examples of prelooped and changing interactions by the three TSSs to enhancers are indicated (*rectangles* and *solid/dotted lines*).

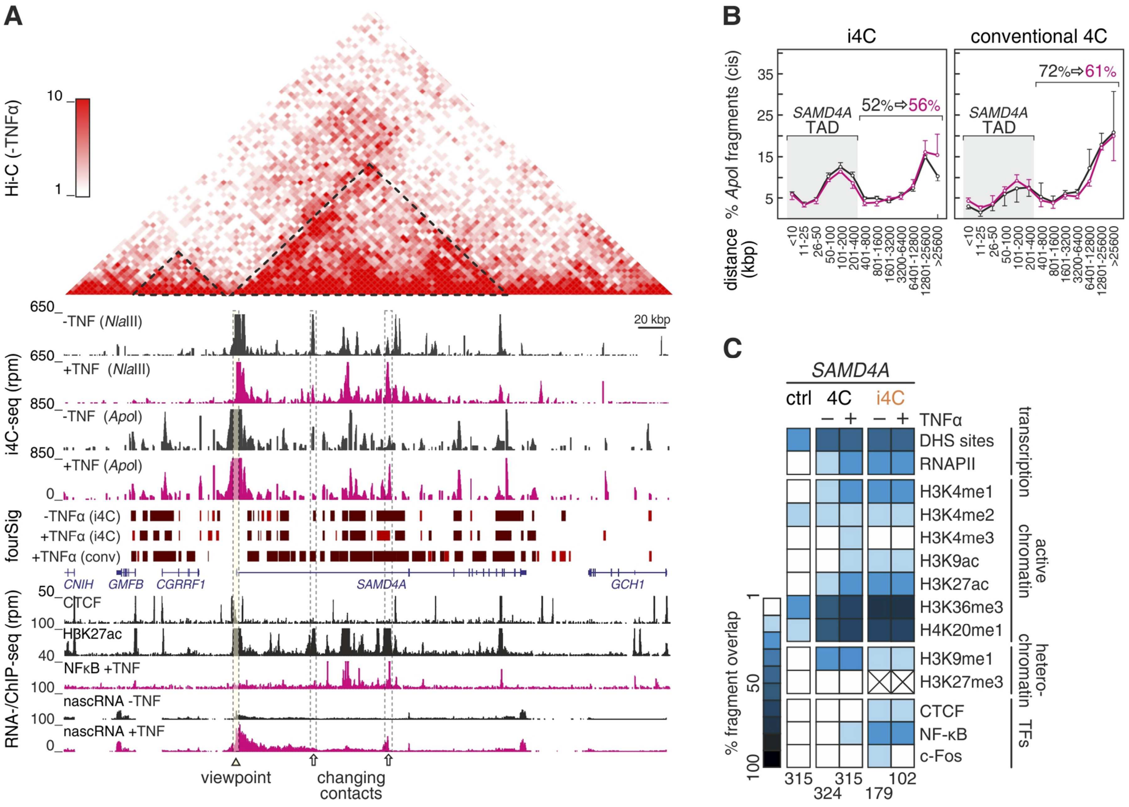

Figure S18. TNF stimulation partially remodels prelooping in *SAMD4A*.

(**A**) i4C-seq was performed in HUVECs stimulated with TNF for 0 (-TNF) or 60 min (+TNF), using NlaIII (*top*) or *Apo*I (*middle*) and the *SAMD4A* TSS as a viewpoint (*triangle*). Browser views show i4C-seq coverage in the *SAMD4A* TAD (*grey rectangle*). RefSeq gene models, ENCODE ChIP-seq data, and nascent RNA-seq data from HUVECs (Caudron-Herger *et al*, 2015) are shown aligned below. *fourSig*: strong (*red*) and intermediate (*brown*) interactions identified in i4C/4C *Apo*I data. *Arrows*: two exemplary changing contacts of the TSS with the *SAMD4A* super-enhancer.

(**B**) Bar plots showing the per cent of *cis*-contacts (*Apo*I fragments; ±SD) made by the *SAMD4A* TSS within and outside its TAD. Results were obtained before (*black line*) and after (*magenta line*) after TNFα stimulation using i4C (*left*; 3 replicates) or conventional 4C (*right*; 2 replicates).

(**C**) Heat map showing the fraction of i4C/4C *SAMD4A cis*-contacts (*Apo*I fragments with >100 rpm) that overlap peaks from DNase I-hypersensitivity (DHS) sites, RNA polymerase (RNAPII), histone marks, and transcription factor (TF) ENCODE datasets (except for the NF-κB ChIP-seq from Papantonis *et al*, 2012) at 0 (-TNF) or 60 min (+TNF) after stimulation. The overlap of randomly-shuffled ("ctrl") fragments serves as a control. The numbers below each heat map indicate the number of *Apo*I fragments analyzed.

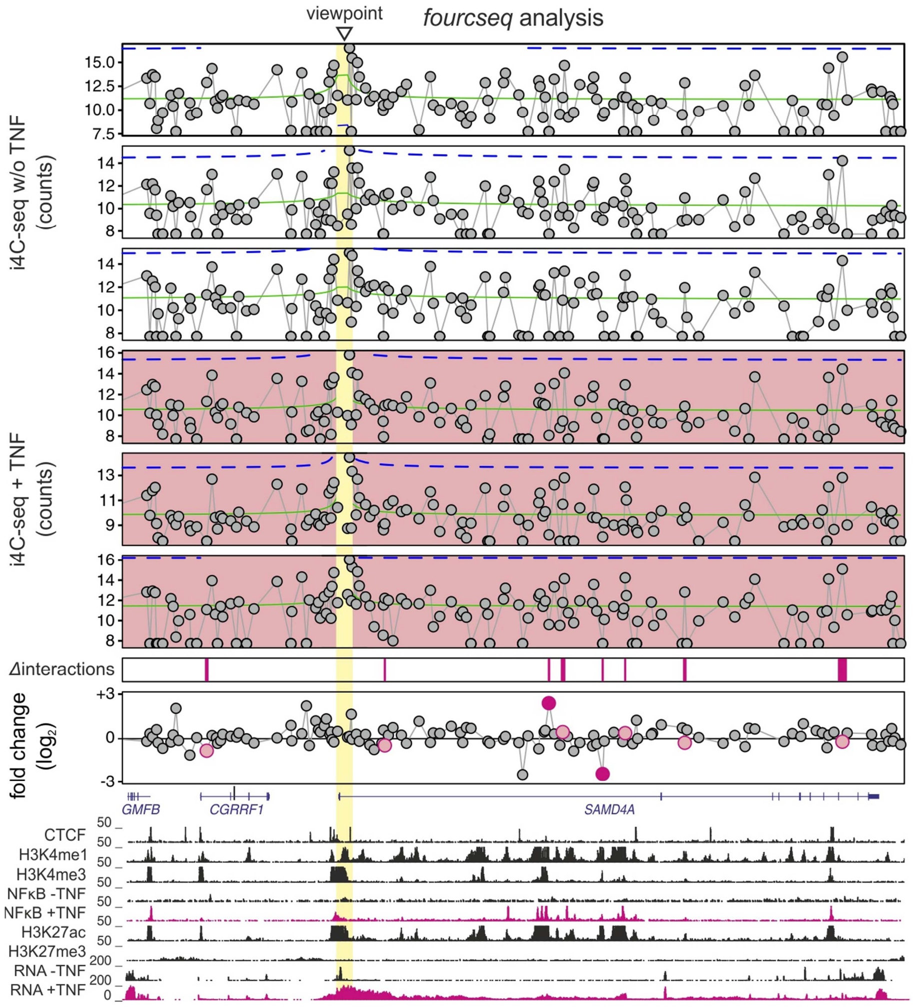

Figure S19. Differential analysis of i4C data upon TNF stimulation.

FourCSeq (Klein *et al*, 2015) was used to perform differential analysis of contacts between 0-min (*white*) and 60-min i4C-seq replicates (*pink*) generated in HUVECs using *Apo*I and the *SAMD4A* TSS as viewpoint (3 replicates each). Some significant differences ("*Δ*interactions") are detected and involve regions differentially bound by NF-κB. HUVEC ChIP-/RNA-seq data (±TNF) are aligned below.

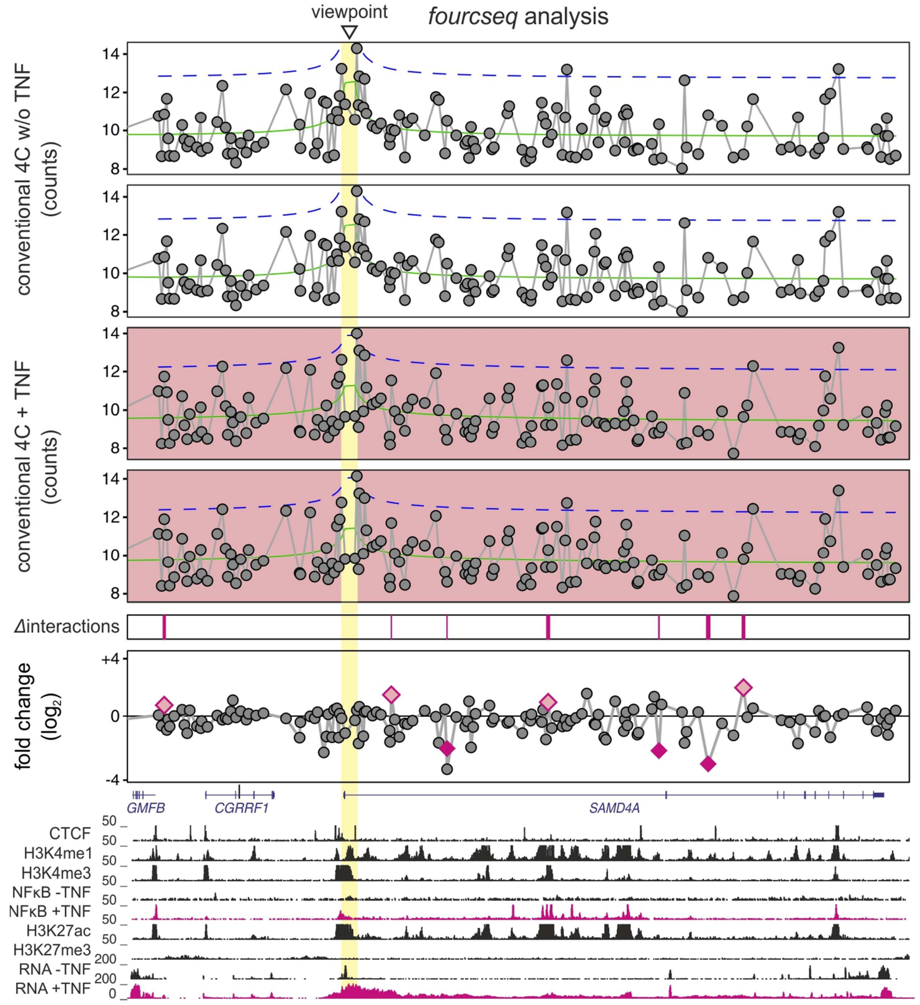

Figure S20. Differential analysis of conventional 4C data upon TNF stimulation.
FourCSeq (Klein *et al*, 2015) was used to perform differential analysis of contacts between 0-min (*white*) and 60-min conventional 4C-seq replicates (*pink*) generated in HUVECs using *Apo*I and the *SAMD4A* TSS as viewpoint (2 replicates each). Some significant differences ("*Δ*interactions") are detected, not always corresponding to regions differentially bound by NF-κB. HUVEC ChIP-/RNA-seq data (±TNF) are aligned below.

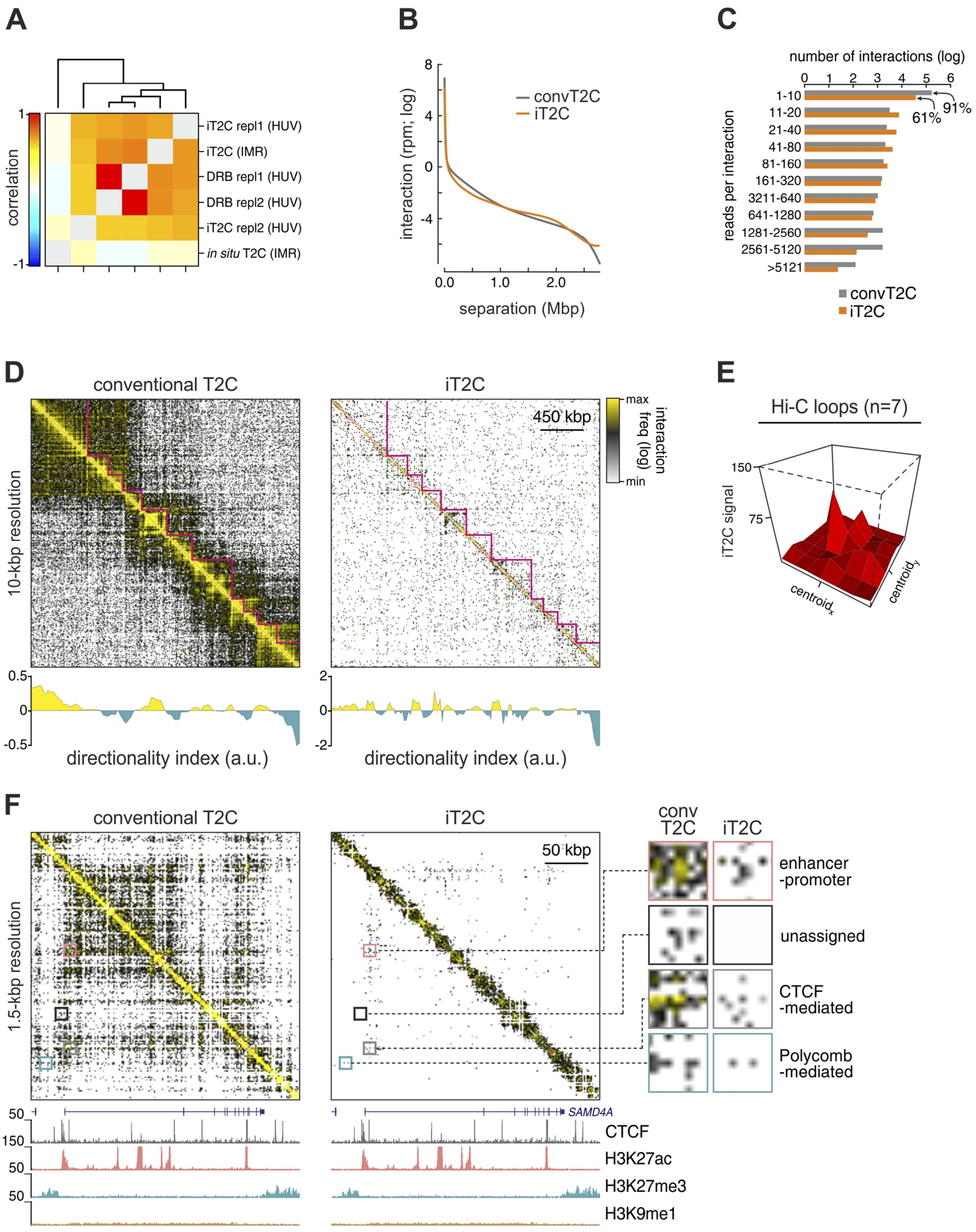

Figure S21. Features of iT2C applied to a 2-8 Mbp human locus on chromosome 14.

(**A**) Heat map showing Spearman's correlations for the different T2C/iT2C replicates.

(**B**) Line plot showing the combined log_{10}-interaction (in rpm) at increasing separations in T2C.

(**C**) Bar plot showing the number of T2C/iT2C *Apo*I fragments ("interactions") binned according to the number of reads (in rpm) mapping to each fragment.

(**D**) Interaction maps from conventional T2C (*left*) and iT2C (*right*) from the 2.8 Mbp around *SAMD4A* were used to calculated the directionality index (Dixon *et al*, 2012) and find domain boundaries (*magenta lines*). Similar partitioning is revealed by either approach.

(**E**) PE-SCAN graph showing the distribution of iT2C at the seven loops contained in the 2.8-Mbp region investigated here (HUVEC-loop centroid$_{x,y}$ positions are from Rao *et al*, 2014).

(**F**) Comparison of conventional and iT2C maps zoomed in the ~250 kbp around *SAMD4A*. *Right*: Exemplary interactions from the two matrices are shown. *Below*: HUVEC ENCODE ChIP-seq data.

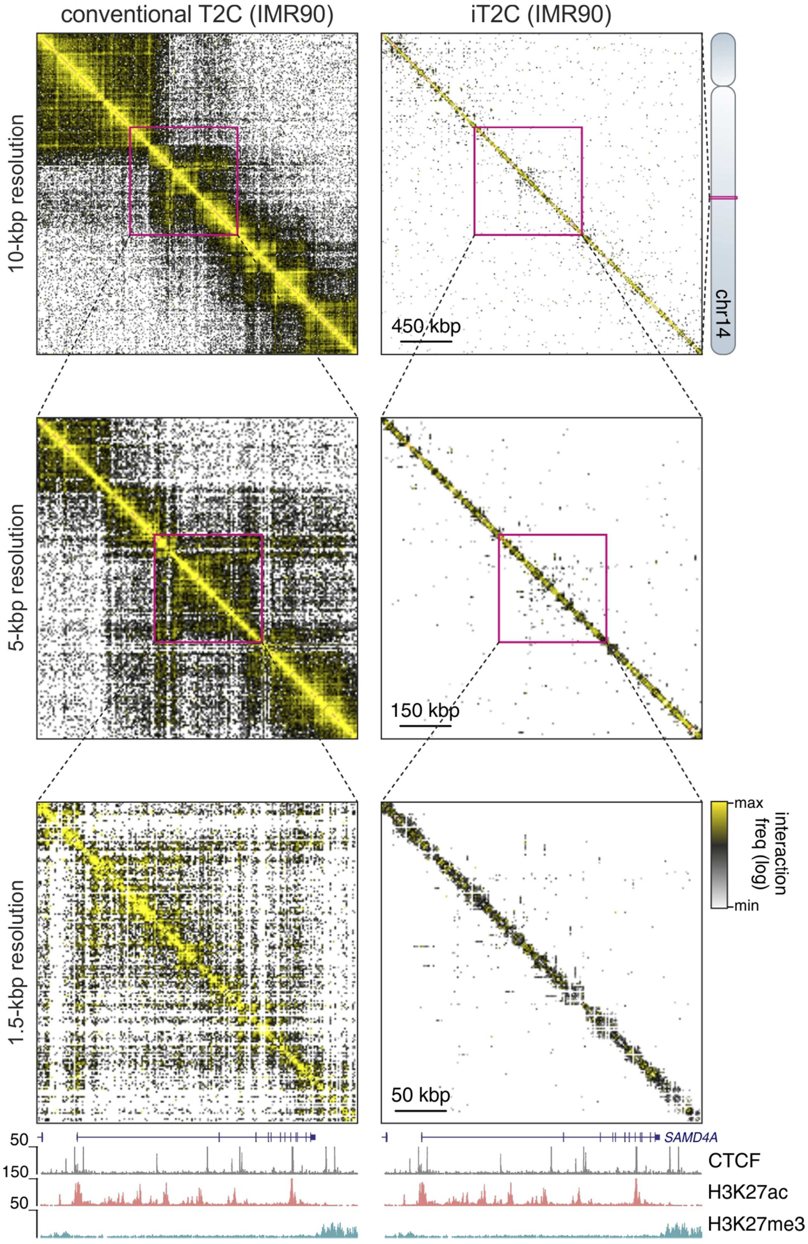

Figure S22. Spatial organization of a 2-8 Mbp human locus in IMR90 cells.
Interaction maps from conventional T2C (*left*) and iT2C (*right*) for the 2.8 Mbp around *SAMD4A* on chromosome 14 (*ideogram*). *Magnifications*: increasingly higher resolution maps are shown. *Bellow*: ENCODE ChIP-seq data are aligned to interactions in the ~250 kbp around *SAMD4A*.

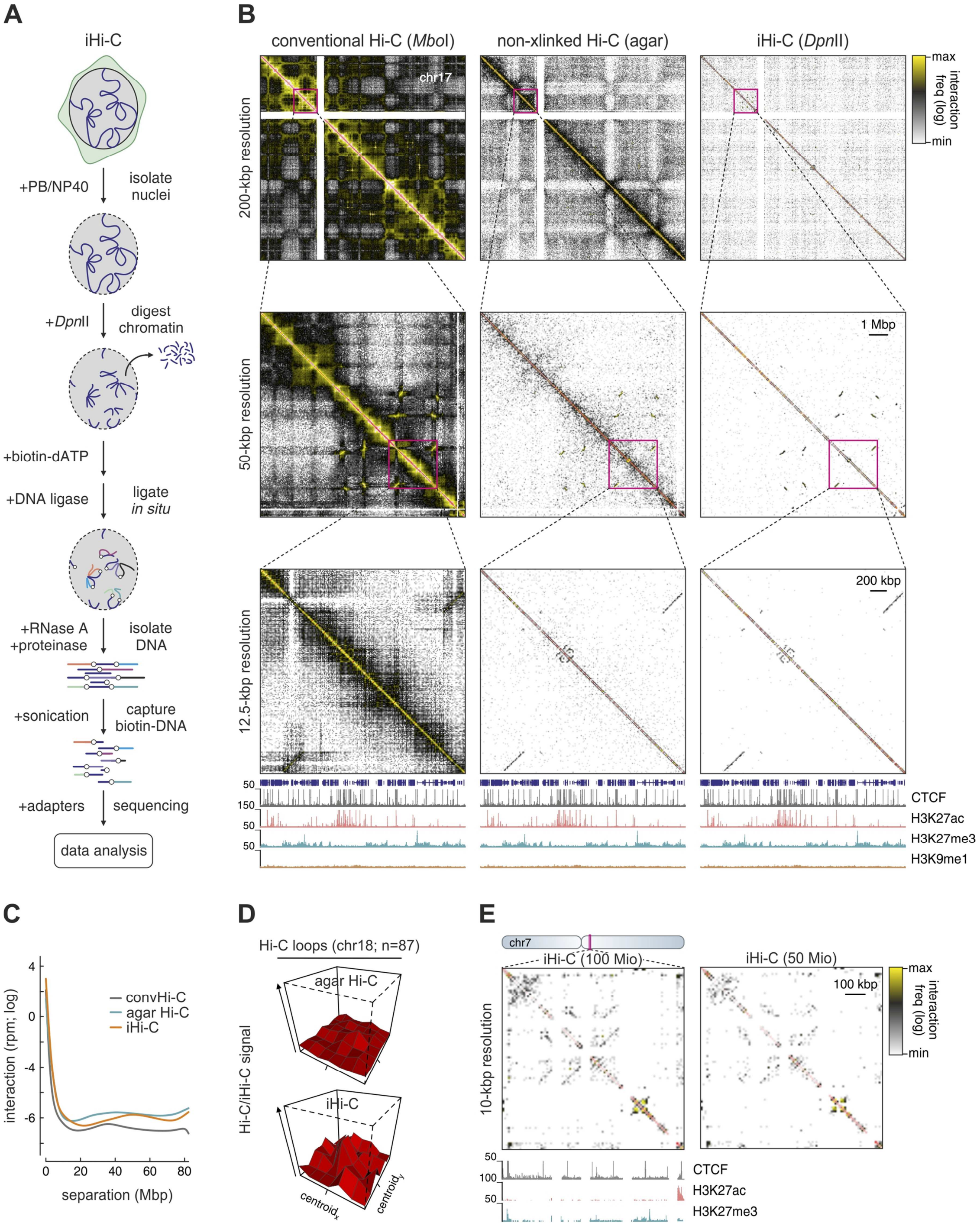

Figure S23. Proof-of-principle iHi-C generated in HUVEC cells.

(**A**) Overview of the adaptation of whole-genome Hi-C in the i3C protocol.

(**B**) Interaction maps for conventional Hi-C (HUVECs: *Hind*III, 500 Mio reads; *left*) and non-crosslinked Hi-C (from lymphoblasts encapsulated in agar plugs, Rao *et al*, 2014: *Mbo*I, ~100 Mio reads; *middle*) and iHi-C (HUVECs: *Apo*I, ~150 Mio reads; *right*) for chromosome 17 were plotted

at 200-, 50-, and 12.5-kbp resolution. *Bottom*: zoom in a 2-Mbp region in the small arm of chr17 aligned to HUVEC ENCODE ChIP-seq data.
(**C**) Line plot showing the combined log_{10}-interaction (in rpm) at increasing separations in iHi-C.
(**D**) PE-SCAN graph showing the distribution of iHi-C signal at the 87 loops detected in Hi-C data from human chr18 (HUVEC-loop $centroid_{x,y}$ positions are from Rao *et al*, 2014).
(**E**) iHi-C interaction maps from HUVECs for a 0.85-Mbp region on the long arm of chromosome 7 (*ideogram*) plotted at 10-kbp resolution using 100 (*left*) or 50 Mio reads (*right*) produce similar profiles. HUVEC ENCODE ChIP-seq data are aligned to the maps (*below*).

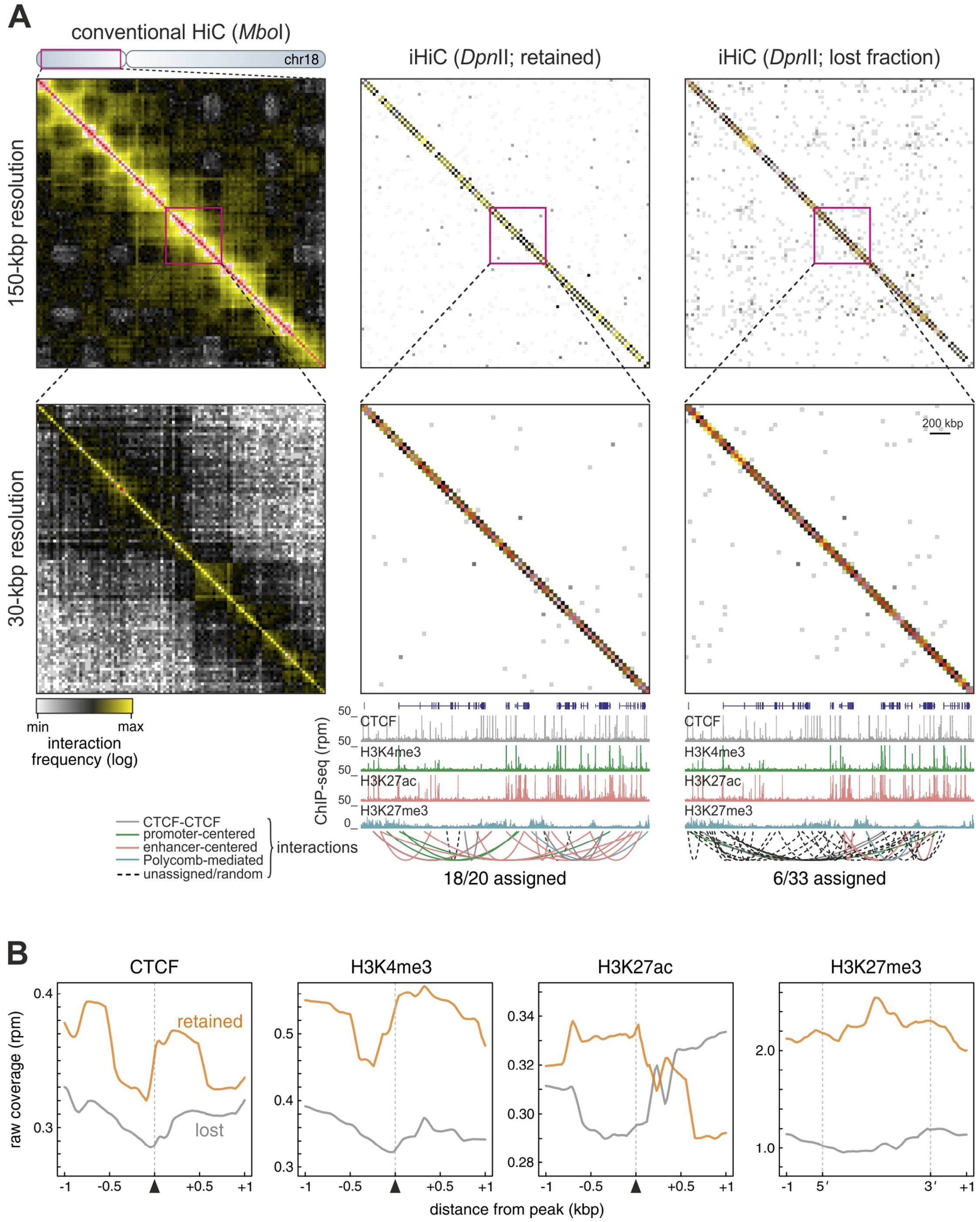

Figure S24. iHi-C generated in the "lost" versus the "retained" fraction of HUVEC cells.
(**A**) Interaction maps for the short arm of chromosome 18 plotted at 150- and 30-kbp resolution from conventional Hi-C (*Mbo*I, 500 Mio reads; *left*) and from iHi-C performed on the "retained" (*Dpn*II, ~200 Mio reads; *middle*) and "lost" chromatin fraction (*Dpn*II, ~200 Mio reads; *right*).

Bottom HUVEC ENCODE ChIP-seq data aligned to a ~2-Mbp region, and the different mapped interactions are shown. The “lost”-fraction iHi-C is largely devoid of interactions.
(**B**) Line plots showing the differences in raw read coverage (in rpm) of “retained” (*orange*) and “lost” iHi-C data (*grey*) around CTCF, H3K4me3, H3K27ac, and H3K27me3 peaks. *Below*: Curved lines (*below*) connect interacting bins and are colour-coded as indicated.

Appendix Tables

Table S1. List of primers used in i3C-/4C-seq experiments [R – reading primer (shown on top), NR – non-reading primer].

Viewpoint	Primer pair (5'-3')	Restriction enzyme	Location (hg19/mm9)
SAMD4A	R: GACGGGTCCGGGTGAATTT<br>NR: CGCAGCCGAACTTTCTTTG	*Apo*I	chr14: 55,032,434-55,033,328
SAMD4A	TCTGTAGACCGAGGGCGGC<br>CAACTCGGACCCTTCACG	*Nla*III	chr14: 55,034,129-55,034,671
EDN1	TTGTTGTGTGCGGGGAATTT<br>GCACTTGGGCTGAAGGATC	*Apo*I	chr6: 12,290,178-12,290,759
BMP4	ACGTGCGGAGGTACTAGAAAG<br>GTCGTTGGGAAAAACTGTGG	*Nla*III	chr14: 54,422,893-54,423,841
CDKN3	CGACACCACCGCTGTCAC<br>ACCCTGCTCCTTCGTCTCTC	*Nla*III	chr14: 54,863,838-54,864,286
CNIH	GCTCCCCGCTCCTCCTCC<br>AAGTGCAAGACAGTGGTGAGAC	*Nla*III	chr14: 54,908,039-54,908,769
TBX5	GACTGAGGTCTCTTGCATAAGG<br>TGAAGAGTTCCCTCCTCTCC	*Nla*III	chr12: 114,846,093-114,846,250
Nanog	CCACCAGCCCTGTGAATTC<br>GGCTCACTTCCTTCTGACTTC	*Apo*I	chr6: 122,657,477-122,657,871
Sox2	CCCAGAAAAATTGTGGTAAAG<br>TCTTTACGTCTGGACAATGG	*Apo*I	chr3: 34,547,092-34,548,290
ZFPM2	GGTCAACTTTTCTTGGCTTGG<br>AAGAGTAGTCCCACGTCAATCG	*Apo*I	chr8:106,329,924-106,332,167

**Illumina adapters used (5'-3'): *Reading primer* – AATGATACGGCGACCACCGAACACTCTTTCCCTACACGACGCTCTTCCGATCT; *Non-reading primer* – CAAGCAGAAGACGGCATACGA

Table S2. List of BACs used for i3C-qPCR normalization.

BAC ID	Chr	Start	End	Genes covered by the BAC
RP11-663M15	6	11287269	11482099	*NEDD9*
RP11-1070E20	6	11687255	11895575	*EDN1* upstream enhancer cluster
RP11-845M8	6	12098076	12292331	*HIVEP1*
RP11-338L10	6	12350362	12513884	*EDN1*
RP11-689G9	6	12498112	12672329	*EDN1* downstream enhancer cluster
RP11-1033M8	2	113232348	113424812	*IL1A*, *IL1B*

Table S3. List of i4C-seq experiments performed and their mapping efficiencies.

Viewpoint	Cell Type	Enzyme	Stimulus	Total reads	Mapped reads	% mapped
SAMD4A	HUVEC replica 1	*Apo*I	-TNF	7543883	3582455	47.49
	HUVEC replica 2	*Apo*I	-TNF	7447826	3107034	41.72
	HUVEC replica 3	*Apo*I	-TNF	18382644	5770332	31.39
	HUVEC replica 4	*Apo*I	-TNF	6864072	2374912	34.60
	HUVEC/2M cells	*Apo*I	-TNF	15110340	5530339	36.60
	HUVEC/1M cells	*Apo*I	-TNF	9781274	2216548	22.66
	HUVEC/0.5M cells	*Apo*I	-TNF	11170290	4041005	36.18
	HUVEC +RNase A	*Apo*I	-TNF	12901124	5885982	45.62
	HUVEC -RNase A	*Apo*I	-TNF	16020312	7513522	46.90
	HUVEC replica 1	*Apo*I	+TNF	8891135	4509100	50.71
	HUVEC replica 2	*Apo*I	+TNF	13362376	6183993	46.28
	HUVEC	*Nla*III	-TNF	6897207	5931643	86.00
	HUVEC	*Nla*III	+TNF	8837391	7927161	89.70
	IMR90	*Apo*I	-TNF	3957052	1318173	33.31
	IMR90	*Apo*I	+TNF	3842205	1397019	36.36
EDN1	HUVEC replica 1	*Apo*I	-TNF	21003319	10980926	52.28
	HUVEC repica 2	*Apo*I	- TNF	7990889	7071894	88.50
CDKN3	HUVEC replica 1	*Apo*I	-TNF	8026058	3029326	37.74
	HUVEC replica 2	*Apo*I	-TNF	13071641	2947164	22.55
	HUVEC replica 3	*Apo*I	-TNF	5909960	2392470	40.48
	HUVEC	*Nla*III	-TNF	9662491	9364579	96.92
	HUVEC	*Nla*III	+TNF	10741289	10327106	96.14
CNIH	HUVEC	*Nla*III	-TNF	8919134	8235445	92.33
	HUVEC	*Nla*III	+TNF	11781791	9810317	83.27
BMP4	HUVEC	*Nla*III	-TNF	12743501	11607337	91.08
	HUVEC	*Nla*III	+TNF	8192590	7247296	88.46
TBX5	HUVEC	*Nla*III	-TNF	9259520	8765025	94.66
	HUVEC	*Nla*III	+TNF	9342151	8658558	92.68
ZFPM2	K562 replica 1	*Apo*I	n/a	4095338	2821520	68.90
	K562 replica 2	*Apo*I	n/a	38356570	31499994	82.12
Nanog	mESCs	*Apo*I	n/a	2465919	2121720	86.04
Sox2	mESCs	*Apo*I	n/a	11834116	7371897	62.29

Table S4. List of iT2C experiments performed and their mapping efficiencies.

Library ID (two replicas each)	Total read pairs	Mapped read pairs (hg19)	% mapped	Read pairs in the 2.8-Mbp subregion
iT2C (HUVEC)	40592135	28412336	69.89	15604832
iT2C+DRB (HUVEC)	47365360	31676149	66.87	9492182
iT2C (IMR90)	44106980	28247260	64.04	13637728
convT2C (HUVEC)	54112557	32032824	59.19	16725700
convT2C (IMR90)	48913930	33298025	68.08	5841754
